# Enhanced robust finite-time passivity for Markovian jumping discrete-time BAM neural networks with leakage delay

**DOI:** 10.1186/s13662-017-1378-9

**Published:** 2017-10-10

**Authors:** C Sowmiya, R Raja, Jinde Cao, G Rajchakit, Ahmed Alsaedi

**Affiliations:** 1 Department of Mathematics, Alagappa University, Karaikudi, 630 004 India; 2 Ramanujan Centre for Higher Mathematics, Alagappa University, Karaikudi, 630 004 India; 3 School of Mathematics, Southeast University, Nanjing, 210096 China; 4 Department of Mathematics, Faculty of Science, Maejo University, Chiang Mai, Thailand; 5 Nonlinear Analysis and Applied Mathematics (NAAM) Research Group, Department of Mathematics, King Abdulaziz University, Jeddah, 21589 Saudi Arabia

**Keywords:** LMIs, Markovian jumping systems, leakage delay, bidirectional associative memory, discrete-time neural networks, passivity and stability analysis

## Abstract

This paper is concerned with the problem of enhanced results on robust finite-time passivity for uncertain discrete-time Markovian jumping BAM delayed neural networks with leakage delay. By implementing a proper Lyapunov-Krasovskii functional candidate, the reciprocally convex combination method together with linear matrix inequality technique, several sufficient conditions are derived for varying the passivity of discrete-time BAM neural networks. An important feature presented in our paper is that we utilize the reciprocally convex combination lemma in the main section and the relevance of that lemma arises from the derivation of stability by using Jensen’s inequality. Further, the zero inequalities help to propose the sufficient conditions for finite-time boundedness and passivity for uncertainties. Finally, the enhancement of the feasible region of the proposed criteria is shown via numerical examples with simulation to illustrate the applicability and usefulness of the proposed method.

## Introduction

Over the past decades, delayed neural networks have found successful applications in many areas such as signal processing, pattern recognition, associative memories and optimization solvers. In such applications quantitative behavior of dynamical systems is an important step for the practical design of neural networks [1]. Therefore, the dynamic characteristics of discrete-time neural networks have been extensively investigated, for example, see [2–12]. The study on neural networks is mostly in the continuous-time setting, but they are often discretized for experimental or computational purposes. Also, neural networks with leakage delay is one of the important types of neural networks. Hence, time delay in the leakage term has a great impact on the dynamics of neural networks. Although, time delay in the stabilizing negative feedback term has a tendency to destabilize a neural network system [13–16], the delay in the leakage term can destroy the stability neural networks. Gopalasamy [17] initially investigated the dynamics of bidirectional associative memory (BAM) network model with leakage delay. Based on this work, authors in [15] considered the global stability for a class of nonlinear systems with leakage delay. Li and Cao discussed the stability of memristive neural networks with both reaction-diffusion term and leakage delay, and some easily checked criteria have been established by employing differential inclusion theory and Jensen’s integral inequality [18].

The (BAM) neural network model, proposed by Kosko [19, 20] is a two-layered nonlinear feedback network model, where the neurons in one layer always interconnect with the neurons in the another layer, while there are no interconnections among neurons in the same layer. In the current scenario, due to its application in many fields, the study of bidirectional associative memory neural networks has attracted the attention of many researchers, and they have studied the stability properties of neural networks and presented various sufficient conditions for asymptotic or exponential stability of the BAM neural networks [4, 15, 21–23].

On the one hand, time delay is one of the main sources of instability, which is encountered in many engineering systems such as chemical processes, long transmission lines in pneumatic systems, networked control systems, *etc*. Over the past years, the study of time delay systems has received considerable attention, and a great number of research results on time delay systems exist in the literature. The stability of time delay systems is a fundamental problem because it is important in the synthesis and analysis of such neural network systems [3, 5, 24, 25]. The exponential stability of stochastic BAM networks with mixed delays was discussed by Lyapunov theory [25].

On the other hand, the theory of passivity was implemented first in circuit analysis and generates increasing interest among the researchers. It is a useful tool in obtaining the stability analysis of both linear and nonlinear systems, especially for high-order systems. It is evidentally true that the passive properties can ideally keep the systems internally stable. Due to its importance and applicability, the problem of passivity analysis for delayed dynamic systems has been investigated, and lots of results have been reported in the literature [26–30]. For instance, in [28] the authors Wu *et al*. derived the passivity condition for discrete-time switched neural networks with various functions and mixed time delays. Moreover, the passivity and synchronization of switched neural networks were investigated in [30], and some delay-dependent as well as delay-independent criteria were provided. In [6, 7], the authors delivered the concept of passivity which is stable or not.

However, using the conventional Lyapunov asymptotic stability theory, it should be mentioned that all these existing studies about the passivity analysis are performed with definition over the infinite-time interval. The concept of finite-time (or short-time) analysis problem was first initiated by Dorato in 1961 [31]. Communication network system, missile system and robot control system are the examples of systems which work in a short-time interval. In the present years, many biologists have been focusing on the transient values of the actual network states. In [32–34], many interesting results for finite-time stability of various types of systems can be found. Recently, an extended finite-time $H_{\infty}$ control problem for uncertain switched linear neutral systems with time-varying delays was investigated in [27], and also the concept of time-varying delays was proposed in [22, 35]. The results for finite-time stabilization of neural networks with discontinuous activations was proposed in [36].

Motivated by the aforementioned discussions, in this paper we focus on the finite-time boundedness and passivity of uncertain discrete-time Markovian jumping BAM neural networks with leakage time-delays. Here we use a new type of LKF to handle the given range of time delay interval together with free weighting matrix approach to derive the main results. Our main contributions are highlighted as follows:

- The finite-time passivity result for discrete-time Markovian jumping uncertain BAM neural networks with leakage delay is proposed for the first time.
- Reciprocally convex combination approach is used to handle the triple summation terms and a new type of zero inequalities is introduced.
- Delay-dependent results for finite-time boundedness and finite-time passivity are derived by using the finite-time stability method and the Lyapunov-Krasovskii functional approach.

The rest of this paper is well organized as follows. Problem formulation and mathematical preliminaries are presented in Section 2. Section 3 gives the main result of this paper, and it also contains the subsection on finite-time boundedness. Robust finite-time passivity is derived in Section 4. Numerical examples are demonstrated in Section 5 to illustrate the effectiveness of the proposed method. Finally, we give the conclusion of this paper in Section 6.

### Notations

The notations in this paper are standard. Throughout this paper, $\mathbb{R}^{n}$ and $\mathbb{R}^{n\times m}$ denote, respectively, the *n*-dimensional Euclidean space and the set of all $n \times m$ real matrices. *I* denotes the identity matrix with appropriate dimensions and $\operatorname{diag}(\cdot)$ denotes the diagonal matrix. $A^{T}$ denotes the transpose of matrix *A*. *k* denotes the set of positive integers. For real symmetric matrices *X* and *Y*, the notation $X \geq Y$ (resp., $X > Y$) means that the matrix $X-Y$ is positive semi-definite (resp., positive definite). $\mathbb{N}= \{1, 2,\ldots, n\}$ and $\|\cdot\|$ stands for the Euclidean norm in $\mathbb{R}^{n}$. $\lambda_{\mathrm{max}}(X)$ (resp., $\lambda _{\mathrm{min}}(X)$) stands for the maximum (resp., minimum) eigenvalue of the matrix *X*. $I_{n}$ and $0_{n}$ represent the identity matrix and zero matrix, respectively. $l_{2}(0, \infty)$ denotes the space of square summable infinite vector sequences. The symbol ∗ within a matrix represents the symmetric term of the matrix.

## Problem formulation and mathematical preliminaries

Let $(\Omega, \mathfrak{F}, \{\mathfrak{F}\}_{t\geq0}, P)$ be a complete probability space with filtration $\{\mathfrak{F}\}_{t\geq 0}$ satisfying the usual condition (*i.e*., it is right continuous and $\mathfrak{F}_{0}$ contains all p-null sets); $E\{\cdot\}$ stands for the mathematical expectation operator with respect to given probability measure *P*. Let $r(k)$, $k\geq0$ be a Markovian chain taking values in a finite space $S = \{1, 2, 3,\ldots, N\}$ with probability transition matrix $\pi = (\pi_{ij})_{N\times N}$ given by

$$ \operatorname{Pr}\bigl[r(k+1) = j|_{r(x)=i}\bigr] = \pi_{ij},\quad j,i \in S, $$

where $\pi\geq0$ ($i, j \in S$) is a transition rate from *i* to *j* and $\sum_{j=1}^{N}\pi_{ij} = 1$, $\forall i \in S$.

Consider the following BAM uncertain discrete-time Markovian jumping neural network with time-varying delays, and leakage delay is described by

1 $$\begin{aligned}& x(k+1) = A\bigl(r(k)\bigr)x(k-\gamma_{1})+B\bigl(r(k)\bigr)f \bigl(y(k)\bigr)+C\bigl(r(k)\bigr)f\bigl(y\bigl(k-\tau (k)\bigr)\bigr)+u(k), \end{aligned}$$

2 $$\begin{aligned}& g^{*}(k) = B_{g^{*}}\bigl(r(k)\bigr)f\bigl(y(k) \bigr)+C_{g^{*}}f\bigl(y\bigl(k-\tau(k)\bigr)\bigr), \end{aligned}$$

3 $$\begin{aligned}& x(k)= \phi(k), \quad\text{for every } k \in [-\tau_{M},0], \\ & y(k+1) = D\bigl(r(k)\bigr)y(k-\gamma_{2})+E\bigl(r(k)\bigr)g \bigl(x(k)\bigr)+F\bigl(r(k)\bigr)g\bigl(x\bigl(k-\sigma (k)\bigr)\bigr)+v(k), \end{aligned}$$

4 $$\begin{aligned}& h(k) = E_{h}\bigl(r(k)\bigr)g\bigl(x(k)\bigr)+F_{h}g\bigl(x \bigl(k-\sigma(k)\bigr)\bigr), \\ & y(k)= \psi(k) \quad\text{for every } k \in [-\sigma_{M},0], \end{aligned}$$

where $x(k), y(k) \in \mathbb{R}^{n}$ is the neural state vector, $v(k)$, $u(k)$ is the exogenous disturbance input vector belonging to $\mathfrak{L}_{2}[0,\infty)$ and $g^{*}(k)$, $h(k)$ is the output vector of the neural network, $f(y(k))$, $g(x(k))$ is the neuron activation function, the positive integer $\tau(k)$, $\sigma (k)$ denotes the time-varying delay satisfying $\tau_{m}\leq\tau (k)\leq\tau_{M}$ and $\sigma_{m}\leq\sigma(k)\leq\sigma_{M}$ for all $k \in N$, where $\tau_{m}$ and $\tau_{M}$, $\sigma_{m}$ and $\sigma_{M}$ are constant positive scalars representing the minimum and maximum delays, respectively.

$$\begin{gathered} A_{i}(k)= A_{i}+\Delta A_{i}(k),\qquad B_{i}(k)= B_{i}+\Delta B_{i}(k), \\ C_{i}(k)= C_{i}+\Delta C_{i}(k),\qquad D_{i}(k)= D_{i}+\Delta D_{i}(k), \\ E_{i}(k)= E_{i}+\Delta E_{i}(k),\qquad F_{i}(k)= F_{i}+\Delta F_{i}(k)\end{gathered} $$

in which $A = \operatorname{diag}\{a_{1},a_{2},\ldots,a_{n}\}$, $D = \operatorname{diag}\{ d_{1},d_{2},\ldots,d_{n}\} $ represent the state feed back coefficient matrix with $|a_{i}|<1$, $|d_{i}|<1$, $B=[b_{ij}]_{n\times n}$, $E=[e_{ij}]_{n\times n}$, $C=[c_{ij}]_{n\times n}$, $F=[f_{ij}]_{n\times n}$, respectively, the connection weights and the delayed connection weights, the initial function $\phi(k)$, $\psi(k)$ is continuous and defined on $[-\tau_{M},0]$, $[-\sigma_{M},0]$. Further the uncertainty parameters are defined as follows:

5 $$ \bigl[\Delta A(k),\Delta B(k),\Delta C(k),\Delta D(k),\Delta E(k),\Delta F(k) \bigr]= M N(k) [M_{a},M_{b},M_{c},M_{d},M_{e},M_{f} ], $$

where $M_{a}$, $M_{b}$, $M_{c}$, $M_{d}$, $M_{f}$, *M* are known constant matrices of appropriate dimensions and $N(k)$ is an unknown time-varying matrix with Lebesgue measurable elements bounded by $N^{T}(k)N(k) \leq I$.

The following assumptions help to complete the main result.

### Assumption I

For any $i = 1, 2, 3, \ldots,n$, there exist constraints $F_{i}^{-}$, $F_{i}^{+}$, $G_{i}^{-}$, $G_{i}^{+}$ such that

$$\begin{gathered} F_{i}^{-} \leq \frac{f_{i}(x_{1})-f_{i}(x_{2})}{x_{1}-x_{2}} \leq F_{i}^{+}\quad \text{for all }x_{1}, x_{2} \in \mathbb{R}, x_{1}\neq x_{2}, \\ G_{i}^{-} \leq \frac{g_{i}(y_{1})-g_{i}(y_{2})}{y_{1}-y_{2}} \leq G_{i}^{+} \quad\text{for all }y_{1}, y_{2} \in \mathbb{R}, y_{1}\neq y_{2}.\end{gathered} $$

For presentation convenience, in the following we denote

$$\begin{gathered} F_{1} = \operatorname{diag} \bigl\{ F_{1}^{-}F_{1}^{+},\ldots,F_{m}^{-}F_{m}^{+} \bigr\} ,\qquad F_{2}=\operatorname{diag} \biggl\{ \frac{F_{1}^{-}+F_{1}^{+}}{2},\ldots, \frac {F_{n}^{-}+F_{n}^{+}}{2} \biggr\} , \\ G_{1} = \operatorname{diag} \bigl\{ G_{1}^{-}G_{1}^{+},\ldots,G_{m}^{-}G_{m}^{+} \bigr\} ,\qquad G_{2}=\operatorname{diag} \biggl\{ \frac{G_{1}^{-}+G_{1}^{+}}{2},\ldots, \frac {G_{n}^{-}+G_{n}^{+}}{2} \biggr\} .\end{gathered} $$

### Remark 2.1

In biologically inspired neural networks, the activation function is usually an abstraction representing the rate of action potential firing in the cell. Non-monotonic functions can be more suitable than other activation functions. In many electronic circuits, the input-output functions of amplifiers may be neither monotonically increasing nor continuously differentiable. The constants are positive, negative or zero in the above assumption. So, the activation function may be non-monotonic and more widespread than usual sigmoid functions and Lipschitz functions. Such conditions are discourteous in qualifying the lower and upper bounds of the activation functions. Therefore, by using the LMI-based technique, the generalized activation function is considered to reduce the possible conservatism.

### Assumption II

The disturbance input vector $v(x)$ and $u(x)$ is time-varying and, for given $v > 0$, $u > 0$ satisfies $v^{T}(x) v(x) \leq v$, $u^{T}(x) u(x) \leq u$.

Before deriving our main results, the following definitions and lemmas will be stated.

### Definition 2.2

[37]

DNN (1), (3) is said to be robustly finite-time bounded with respect to $(\eta_{1}, \eta_{2}, \eta, \chi, L, Q, L_{1}, Q_{1}, u, v)$, where $0 < \eta_{1} < \eta_{2} < \eta< \chi$ and $L, L_{1} > 0$, if

$$ x^{T}(k_{1})L x(k_{1})+y^{T}(k_{2})L_{1} y(k_{2})\leq\eta\quad \Rightarrow\quad x^{T}(k_{3})L x(k_{3})+y^{T}(k_{4})L_{1} y(k_{4}) \leq\chi, $$

∀ $k_{1} \in \{-\tau_{M}, -\tau_{M}+1,\ldots, 0 \} $, $k_{2} \in \{-\sigma_{M}, -\sigma_{M}+1,\ldots, 0 \} $, $k_{4}=k_{3} \in \{1, 2,\ldots, N \}$ holds for any nonzero $v(x)$, $u(x)$ satisfying Assumption II.

### Definition 2.3

[37]

DNN (1), (3) with output (2), (4) is said to be robustly finite-time passive with respect to $(\eta_{1}, \eta_{2}, \eta, \chi, L, Q, L_{1}, Q_{1}, \gamma, \tilde{\gamma }_{1}, u, v)$, where $0 < \eta_{1} < \eta_{2} < \eta< \chi, \omega , \tilde{\omega}$ is a prescribed positive scalar and $L > 0$ and $L_{1} > 0$, iff DNN (1), (3) with output (2), (4) is robustly finite-time bounded with respect to $(\eta_{1}, \eta _{2}, \eta, \chi, L, Q, L_{1}, Q_{1}, u, v)$ and under the zero initial condition the output $G(k)$, $H(k)$ satisfies

$$ 2 \Biggl[\sum_{k=0}^{N}g^{{*}^{T}}(k) u(k)+ \sum_{k=0}^{N}h^{T}(k) v(k) \Biggr] \geq \gamma \Biggl[\sum_{k=0}^{T}u^{T}(k) u(k)+ \sum_{k=0}^{T_{1}}v^{T}(k) v(k) \Biggr] $$

for any nonzero $v(x)$, $u(x)$ satisfying Assumption II.

### Remark 2.4

The concept of finite-time passivity is different from that of usual passivity. If the states in the system exceed their recommended bounds, it is usual passivity. Here, in this paper, Assumption II and Definition 2.2 should be in the given bounds, which helps to prove the finite-time passivity in the main result.

### Lemma 2.5

[12]

*For any symmetric constant matrix* $Z \in \mathbb{R}^{n \times n}$, $Z \geq0$, *two scalars* $\tau_{m}$ *and* $\tau_{M}$ *satisfying* $\tau_{m} \leq\tau_{M}$, *and a vector*-*valued function* $\eta{k} = x(k+1)-x(k)$ ($k \in\mathbb{Z}^{+}$), *we have*

$$\begin{gathered} \mathrm{(i)}\quad {-}\sum_{i=k-\tau_{M}}^{k-\tau_{m}-1} \eta^{T}(i)Z\eta(i) \leq \frac{-1}{(\tau_{M}-\tau_{m})}\sum _{i=k-\tau_{M}}^{k-\tau _{m}-1}\eta^{T}(i)Z \sum _{i=k-\tau_{M}}^{k-\tau_{m}-1}\eta(i); \\ \mathrm{(ii)}\quad {-}\sum_{j=-\tau_{M}}^{-\tau_{m}-1}\sum _{i=k+j}^{k-1}\eta ^{T}(i)Z\eta(i)\\ \phantom{\mathrm{(ii)}}\qquad \leq \frac{-2}{(\tau_{M}-\tau_{m})(\tau_{M}+\tau _{m}+1)}\sum_{j=-\tau_{M}}^{-\tau_{m}-1}\sum _{i=k+j}^{k-1}\eta ^{T}(i)Z \sum _{j=-\tau_{M}}^{-\tau_{m}-1}\sum_{i=k+j}^{k-1} \eta ^{T}(i)\eta(i).\end{gathered} $$

### Lemma 2.6

[38]

*Let* $g_{1}, g_{2},\ldots,g_{N}: \mathbb{R}^{m} \rightarrow\mathbb{R}$ *have positive values in an open subset* $\mathbb{D}$ *of* $\mathbb{R}^{m}$. *Then the reciprocally convex combination of* $g_{i}$ *over* $\mathbb{D}$ *satisfies*

$$ \min_{\{\alpha_{i}|\alpha_{i} >0, \sum_{i}\alpha_{i}=1\}}\sum_{i} \frac{1}{\alpha_{i}}g_{i}(t) = \sum_{i}g_{i}(t)+ \max_{h_{ij}(t)}\sum_{i \neq j}h_{ij}(t) $$

*subject to*

$$ \left\{h_{ij}: \mathbb{R}^{m} \rightarrow\mathbb{R}, h_{ij}(t) = h_{ji}(t), \begin{pmatrix} g_{i}(t)& h_{i,j}(t)\\ h_{i,j}(t)& g_{j}(t) \end{pmatrix} \right\}. $$

### Remark 2.7

There are two main methods to find lower bounds. The first one is based on Moon’s inequality. The second method is the so-called reciprocally convex combination lemma, and this approach also helps to reduce the number of decision variables. The conservatism induced by these two inequalities is independent. While, in some cases, such as stability conditions resulting from the application of Jensen’s inequality, the reciprocally convex combination lemma is in general more conservative than Moon’s inequality. Also, note that the reciprocally convex combination approach is successfully applied on double summation terms.

## Robust finite-time boundedness

The main concern in this subsection is that the sufficient conditions for the finite boundedness of DNN (1), (3) and the LMI-based robust conditions will be derived using the Lyapunov technique.

### Theorem 3.1

*Under Assumptions* I *and* II, *for given scalars* $\mu>1$, $\rho>1$, $\tau_{m}$, $\tau_{M}$, $\sigma_{m}$, $\sigma_{M}$, *DNN model* (1), (3) *is robustly finite*-*time bounded with respect to* $(\eta_{1}, \eta_{2}, \eta, \chi, L, Q, L_{1}, Q_{1}, u, v)$ *if there exist symmetric positive definite matrices* $P_{1i}$, $P_{2i}$, *W*, $W_{1}$, $R_{1}$, *R*, $S_{1}$, $S_{2}$, $S_{3}$, $S_{4}$, $Z_{1}$, $Z_{2}$, $Z_{3}$, $Z_{4}$, *matrices* $U_{1}$, $U_{2}$, $U_{3}$, $U_{4}$, $U_{5}$, $U_{6}$, $U_{7}$, $U_{8}$, *positive diagonal matrices* $H_{1}$, $H_{2}$, $H_{3}$, $H_{4}$ *and positive scalars* $\lambda^{P_{1}}$, $\lambda^{P_{2}}$, $\lambda _{P_{1}}$, $\lambda_{P_{2}}$, $\lambda_{W_{3}}$, $\lambda_{W_{4}}$, $\lambda_{R_{3}}$, $\lambda_{R_{4}}$, $\lambda_{S_{1}}$, $\lambda _{S_{2}}$, $\lambda_{S_{3}}$, $\lambda_{S_{4}}$, $\lambda_{Z_{1}}$, $\lambda_{Z_{2}}$, $\lambda_{Z_{3}}$, $\lambda_{Z_{4}}$, *ϵ*, $\epsilon_{1}$, $P_{1i}=P_{1}(r_{k})$, $P_{2i}=P_{2}(r_{k})$, $\bar {P}_{1i}= \sum_{j=1}^{N}\Pi_{ij}P_{1j}$, $\bar{P}_{2i}= \sum_{j=1}^{N}\Pi_{ij}P_{2j}$ *such that the following LMIs hold for* $r=1, 2, 3, 4$:

6 $$\begin{aligned}& \begin{pmatrix} \tilde{\Pi}_{r}& \tilde{\Pi}_{1}^{T}P_{1i}& \hat{\sigma}\tilde {\Pi}_{2}^{T}Z_{1}& \frac{\check{\sigma}}{2}\tilde{\Pi }_{2}^{T}Z_{2}& 0& \epsilon\tilde{M}^{T}_{ABC}\\ *& -P_{1i}& 0& 0& M& 0\\ *& *& -Z_{1}& 0& M& 0\\ *& *& *& -Z_{2}& M& 0\\ *& *& *& *& -\epsilon I& 0\\ *& *& *& *& *& -\epsilon I \end{pmatrix} < 0 ,\quad r=1,2, \end{aligned}$$

7 $$\begin{aligned}& \begin{pmatrix} \breve{\Pi}_{r}& \breve{\Pi}_{1}^{T}P_{2i}& \hat{\tau}\breve{\Pi }_{2}^{T}Z_{3}& \frac{\check{\tau}}{2}\breve{\Pi}_{2}^{T}Z_{4}& 0& \epsilon^{*}\breve{M}^{T}_{DEF}\\ *& -P_{2i}& 0& 0& M& 0\\ *& *& -Z_{1}& 0& M& 0\\ *& *& *& -Z_{2}& M& 0\\ *& *& *& *& -\epsilon^{*} I& 0\\ *& *& *& *& *& -\epsilon^{*} I \end{pmatrix} < 0 ,\quad r=3,4, \end{aligned}$$

8 $$\begin{aligned}& \begin{pmatrix} Z_{1}& U_{1}\\ *& Z_{1} \end{pmatrix} \geq0, \qquad \begin{pmatrix} Z_{2}& U_{2}\\ *& Z_{2} \end{pmatrix} \geq0, \qquad \begin{pmatrix} Z_{3}& U_{3}\\ *& Z_{3} \end{pmatrix} \geq0, \qquad \begin{pmatrix} Z_{4}& U_{4}\\ *& Z_{4} \end{pmatrix} \geq0, \end{aligned}$$

9 $$\begin{aligned}& \begin{pmatrix} S_{1}& U_{5}\\ *& S_{1} \end{pmatrix} \geq0, \qquad \begin{pmatrix} S_{2}& U_{6}\\ *& S_{2} \end{pmatrix} \geq0, \qquad \begin{pmatrix} S_{3}& U_{7}\\ *& S_{3} \end{pmatrix} \geq0, \qquad \begin{pmatrix} S_{4}& U_{8}\\ *& S_{4} \end{pmatrix} \geq0, \end{aligned}$$

10 $$\begin{aligned}& \begin{gathered}\lambda^{P_{1}}I \leq P_{1}\leq\lambda_{P_{1}} I,\qquad \lambda ^{P_{2}}I \leq P_{2}\leq\lambda_{P_{2}} I,\qquad 0< W_{3}\leq\lambda W_{3} I, \\ 0< W_{4}\leq \lambda W_{4} I, \qquad 0< R_{3}\leq\lambda R_{3} I,\qquad 0< R_{4}\leq\lambda R_{4} I, \\ 0 < S_{r1} \leq\lambda_{S_{r}}I, \qquad 0 < Z_{r} \leq\lambda_{Z_{r}}I,\end{gathered} \end{aligned}$$

11 $$\begin{aligned}& \psi_{1}\eta_{1}+\psi_{2}\kappa+ \lambda_{Q_{1}}u+\psi_{3}\eta _{2}+ \psi_{4}\kappa_{1}+\lambda_{Q}v < M^{*}\chi\mu^{-N}\rho ^{-N}, \end{aligned}$$

*where*

$$\begin{aligned}& \begin{aligned}\tilde{\Pi}_{r}={}& \begin{pmatrix} \tilde{\Pi}_{1,1,i}& 0& 0& 0& 0& 0& 0& 0& 0& 0\\ *& \tilde{\Pi}_{2,2,i}& 0& 0& 0& G_{2}H_{1}& 0& \tilde{\Pi}_{r2}& \tilde{\Pi}_{r3}& 0\\ *& *& \tilde{\Pi}_{3,3,i}& \tilde{\Pi}_{3,4,i}& \mu^{\sigma _{m}+1}U_{1}^{T}& 0& 0& 0& 0& 0\\ *& *& *& \tilde{\Pi}_{4,4,i}& \tilde{\Pi}_{4,5,i}& 0& G_{2}H_{2}& 0& 0& 0\\ *& *& *& *& \tilde{\Pi}_{5,5,i}& 0& 0& 0& 0& 0\\ *& *& *& *& *& \tilde{\Pi}_{6,6,i}& 0& 0& 0& 0\\ *& *& *& *& *& *& \tilde{\Pi}_{7,7,i}& 0& 0& 0\\ *& *& *& *& *& *& *& \tilde{\Pi}_{8,8,i}& \tilde{\Pi}_{8,9,i}& 0\\ *& *& *& *& *& *& *& *& \tilde{\Pi}_{9,9,i}& 0\\ *& *& *& *& *& *& *& *& *& -Q \end{pmatrix} ,\\& r=1,2,\end{aligned} \\& \begin{aligned}\breve{\Pi}_{r}= {}& \begin{pmatrix} \breve{\Pi}_{1,1,i}& 0& 0& 0& 0& 0& 0& 0& 0& 0\\ *& \breve{\Pi}_{2,2,i}& 0& 0& 0& F_{4}H_{3}& 0& \breve{\Pi}_{r2}& \breve{\Pi}_{r3}& 0\\ *& *& \breve{\Pi}_{3,3,i}& \breve{\Pi}_{3,4,i}& \rho^{\tau _{m}+1}U_{3}^{T}& 0& 0& 0& 0& 0\\ *& *& *& \breve{\Pi}_{4,4,i}& \breve{\Pi}_{4,5,i}& 0& G_{4}H_{4}& 0& 0& 0\\ *& *& *& *& \breve{\Pi}_{5,5,i}& 0& 0& 0& 0& 0\\ *& *& *& *& *& \breve{\Pi}_{6,6,i}& 0& 0& 0& 0\\ *& *& *& *& *& *& \breve{\Pi}_{7,7,i}& 0& 0& 0\\ *& *& *& *& *& *& *& \breve{\Pi}_{8,8,i}& \breve{\Pi}_{8,9,i}& 0\\ *& *& *& *& *& *& *& *& \breve{\Pi}_{9,9,i}& 0\\ *& *& *& *& *& *& *& *& *& -Q_{1} \end{pmatrix} ,\\ &r=3,4,\end{aligned} \\& \tilde{\Pi}_{2,2,i} = (1+\hat{\sigma})W+\hat{\sigma} \bigl(S_{1}+\mu ^{\sigma_{m}+1}U_{6}\bigr)+ \frac{\hat{\sigma}}{2}S_{2}-\mu^{\sigma _{m}+1}\hat{\sigma}^{2}Z_{2}- \mu P_{1i}-G_{1}H_{1}, \\& \tilde{\Pi}_{12} = \mu^{\sigma_{m}+1} \hat{\sigma}U_{2},\qquad \tilde{\Pi}_{13} = \mu^{\sigma_{m}+1}\hat{\sigma}Z_{2},\qquad \tilde{\Pi}_{3,3,i} = \mu^{\sigma_{m}+1}(U_{5}-Z_{1}), \\& \tilde{\Pi}_{3,4,i} = \mu^{\sigma_{m}+1}\bigl(Z_{1}-U_{1}^{T} \bigr), \qquad \tilde{\Pi}_{4,4,i} = \mu^{\sigma_{m}+1}\operatorname{sym}(U_{1}-Z_{1})- \mu ^{\sigma_{m}}W-G_{1}H_{2}, \\& \tilde{\Pi}_{4,5,i} = \mu^{\sigma_{m}+1}\bigl(Z_{1}-U_{1}^{T} \bigr), \qquad \tilde{\Pi}_{5,5,i} = -\mu^{\sigma_{m}+1}(Z_{1}+U_{5}),\qquad \tilde{\Pi}_{6,6,i} = (1+\hat{\sigma})R-H_{1}, \\& \tilde{\Pi}_{7,7,i} = -\mu^{\sigma_{M}+1}R-H_{2},\qquad \tilde{\Pi}_{8,8,i} = -\mu^{\sigma_{m}+1}\biggl(Z_{2}+ \frac{1}{\hat {\sigma}}U_{6}\biggr), \\& \tilde{\Pi}_{8,9,i} = -\mu^{\sigma_{m}+1}\biggl(U_{2}^{T}- \frac {1}{\hat{\sigma}}U_{6}\biggr), \qquad\tilde{\Pi}_{9,9,i} = - \mu^{\sigma_{m}+1}\biggl(Z_{2}+\frac{1}{\hat {\sigma}}U_{6} \biggr), \\& \tilde{\Pi}_{22} = \mu^{\sigma_{m}+1} \hat{\sigma}Z_{2},\qquad \tilde{\Pi}_{23} = \mu^{\sigma_{m}+1}\hat{\sigma}U_{2}^{T}, \\& \breve{\Pi}_{2,2,i} = (1+\hat{\tau})W_{1}+\hat{\tau} \bigl(S_{3}+\rho ^{\tau_{m}+1}U_{8}\bigr)+ \frac{\hat{\tau}}{2}S_{4}-\rho^{\tau _{m}+1}\hat{\tau}^{2}Z_{4}- \rho P_{2i}-F_{3}H_{3}, \\& \breve{\Pi}_{32} = \rho^{\tau_{m}+1} \hat{\tau}U_{4},\qquad \breve{\Pi}_{33} = \rho^{\tau_{m}+1}\hat{\tau}Z_{4},\qquad \breve{\Pi}_{3,3,i} = \rho^{\tau_{m}+1}(U_{7}-Z_{3}), \\& \breve{\Pi}_{3,4,i} = \rho^{\tau_{m}+1}\bigl(Z_{3}-U_{3}^{T} \bigr), \qquad \breve{\Pi}_{4,4,i} = \rho^{\tau_{m}+1}\operatorname{sym}(U_{3}-Z_{3})- \rho^{\tau _{m}}W_{1}-G_{3}H_{4}, \\& \breve{\Pi}_{4,5,i} = \rho^{\tau_{m}+1}\bigl(Z_{3}-U_{3}^{T} \bigr), \qquad \breve{\Pi}_{5,5,i} = -\rho^{\tau_{m}+1}(Z_{3}+U_{7}),\qquad \breve{\Pi}_{6,6,i} = (1+\hat{\tau})R_{1}-H_{3}, \\& \breve{\Pi}_{7,7,i} = -\rho^{\tau_{M}}R_{1}-H_{4}, \qquad \breve{\Pi}_{8,8,i} = -\rho^{\tau_{m}+1}\biggl(Z_{4}+ \frac{1}{\hat{\tau }}U_{8}\biggr), \\& \breve{\Pi}_{8,9,i} = -\rho^{\tau_{m}+1}\biggl(U_{4}^{T}- \frac{1}{\hat {\tau}}U_{8}\biggr), \qquad \breve{\Pi}_{9,9,i} = - \rho^{\tau_{m}+1}\biggl(Z_{4}+\frac{1}{\hat{\tau }}U_{8} \biggr), \\& \breve{\Pi}_{42} = \rho^{\tau_{m}+1} \hat{\tau}Z_{4},\qquad \breve{\Pi}_{43} = \rho^{\tau_{m}+1}\hat{\tau}U_{4}^{T}, \\& \tilde{\Pi}_{1} = [\begin{matrix}A & 0 & 0 & 0 & 0 & B & C & 0 & 0 & I \end{matrix}], \\& \tilde{\Pi}_{2} = [\textstyle\begin{array}{c@{\quad}c@{\quad}c@{\quad}c@{\quad}c@{\quad}c@{\quad}c@{\quad}c@{\quad}c@{\quad}c@{\quad}c}A-I & 0 & 0 & 0 & 0 & 0 & B & C & 0 & 0 & I \end{array}\displaystyle ] \\& \breve{\Pi}_{3} = [\begin{matrix}D & 0 & 0 & 0 & 0 & E & F & 0 &0 & I \end{matrix}], \\& \breve{\Pi}_{4} = [\textstyle\begin{array}{c@{\quad}c@{\quad}c@{\quad}c@{\quad}c@{\quad}c@{\quad}c@{\quad}c@{\quad}c@{\quad}c}D-I & 0 & 0 & 0 & 0 & E & F & 0 & 0 & I\end{array}\displaystyle ], \\& \tilde{M}_{ABC} = [\begin{matrix}M_{A} & 0 & 0 & 0 & 0& M_{B} & M_{C} & 0 & 0 & 0 \end{matrix}],\\& \breve{M}_{DEF} = [\begin{matrix}M_{D} & 0 & 0 & 0 & 0 &M_{E} & M_{F} & 0 & 0 & 0 \end{matrix}], \\& \hat{\tau} = \tau_{M}-\tau_{m}, \qquad \check{\tau} = ( \tau_{M}-\tau_{m}) (\tau_{M}+ \tau_{m}+1), \\& \hat{\sigma} = \sigma_{M}-\sigma_{m}, \qquad \check{ \sigma} = (\sigma_{M}-\sigma_{m}) (\sigma_{M}+ \sigma _{m}+1), \\& \hat{f} = \max_{1 \leq i \leq n} \bigl\{ \big|F_{i}^{-}\big|, \big|F_{i}^{+}\big| \bigr\} , \qquad \hat{g} = \max _{1 \leq i \leq n} \bigl\{ \big|G_{i}^{-}\big|, \big|G_{i}^{+}\big| \bigr\} , \\& \begin{aligned}\psi_{1} ={}& \lambda_{P_{1}}+ \biggl(\mu^{\sigma_{M}-1}\sigma _{M}+\mu^{\sigma_{M}}\frac{\hat{\sigma}(\sigma_{M}+\sigma _{m}-1)}{2} \biggr) \bigl(\lambda W_{3}+\hat{f}^{2}\lambda_{R_{3}}\bigr)+\lambda _{S_{11}}\mu^{\sigma_{m}-1}\frac{\check{\sigma}}{2} \\ &+\lambda_{S_{31}}\mu^{\sigma_{M}-1} \biggl[\biggl( \frac {1}{12}\sigma_{m}(\sigma_{m}+1) (2 \sigma_{M}+1) -\sigma_{m}(\sigma_{m}+1) (2 \sigma_{m}+1)\biggr)-\frac{\check{\sigma }}{4} \biggr],\end{aligned} \\& \begin{aligned}\psi_{2} ={} &\lambda_{Z_{1}}\mu^{\sigma_{M}-1} \frac{\hat{\sigma }\check{\sigma}}{2}+\lambda_{Z_{2}} \\ & +\mu^{\sigma_{m}-1}\check {\sigma} \biggl[\biggl(\frac{1}{24}\sigma_{m}(\sigma_{m}+1) (2 \sigma_{M}+1) -\sigma_{m}(\sigma_{m}+1)(2\sigma_{m}+1)\biggr)-\frac{\check{\sigma}}{8} \biggr],\end{aligned} \\& \begin{aligned}\psi_{3} ={}& \lambda_{P_{2}}+ \biggl(\rho^{\tau_{M}-1} \tau_{M}+\rho ^{\tau_{M}}\frac{\hat{\tau}(\tau_{M}+\tau_{m}-1)}{2} \biggr) \bigl(\lambda W_{4}+\hat{g}^{2}\lambda_{R_{4}}\bigr)+ \lambda_{S_{21}}\mu ^{\tau_{m}-1}\frac{\check{\tau}}{2} \\ &+\lambda_{S_{41}}\rho^{\tau_{M}-1} \biggl[\biggl( \frac{1}{12}\tau _{m}(\tau_{m}+1) (2 \tau_{M}+1) -\tau_{m}(\tau_{m}+1) (2 \tau_{m}+1)\biggr)-\frac{\check{\tau}}{4} \biggr],\end{aligned} \\& \begin{aligned}\psi_{4} ={}& \lambda_{Z_{3}}\rho^{\tau_{M}-1} \frac{\hat{\tau }\check{\tau}}{2}+\lambda_{Z_{4}} \\ &+\rho^{\tau_{m}-1}\check{\tau } \biggl[\biggl(\frac{1}{24}\tau_{m}(\tau_{m}+1) (2 \tau_{M}+1) -\tau_{m}(\tau_{m}+1)(2\tau_{m}+1)\biggr)-\frac{\check{\tau}}{8} \biggr].\end{aligned} \end{aligned}$$

### Proof

To prove the required results, the following LKF for finite-time passivity BAM DNN model (1)-(4) is considered:

12 $$ V(k) = \sum_{i=1}^{5}V_{i}(k), $$

where

$$\begin{aligned}& \begin{aligned}V_{1i}(k)={}& V_{11}(k)+V_{12}(k) \\ ={}& x^{T}(k)P_{1i}x(k)+y^{T}(k)P_{2i} y(k),\end{aligned} \\& \begin{aligned}V_{2i}(k)={}& V_{21}(k)+V_{22}(k) \\ ={}& \sum_{i=k-\sigma(k)}^{k-1}\mu^{k-i-1} x^{T}(i)W x(i)+ \sum_{j=-\sigma_{M}+1}^{-\sigma_{M}} \sum_{i=k+j}^{k-1}\mu^{k-i-1} x^{T}(i)W x(i) \\ & +\sum_{i=k-\tau(k)}^{k-1}\rho^{k-i-1} y^{T}(i)W_{1} y(i)+ \sum_{j=-\tau_{M}+1}^{-\tau_{M}} \sum_{i=k+j}^{k-1}\rho^{k-i-1} y^{T}(i)W_{1} y(i),\end{aligned} \\& \begin{aligned}V_{3i}(k) ={}& V_{31}(k)+V_{32}(k) \\ ={}& \sum_{i=k-\sigma(k)}^{k-1}\mu^{k-i-1} g^{T}\bigl(x(i)\bigr)R g\bigl(x(i)\bigr)+ \sum _{j=-\sigma_{M}+1}^{-\sigma_{M}} \sum_{i=k+j}^{k-1} \mu^{k-i-1} g^{T}\bigl(x(i)\bigr)R g\bigl(x(i)\bigr) \\ & +\sum_{i=k-\tau(k)}^{k-1}\rho^{k-i-1} f^{T}\bigl(y(i)\bigr)R_{1} f\bigl(y(i)\bigr)+ \sum _{j=-\tau_{M}+1}^{-\tau_{M}} \sum_{i=k+j}^{k-1} \rho ^{k-i-1} f^{T}\bigl(y(i)\bigr)R_{1} f\bigl(y(i) \bigr),\end{aligned} \\& \begin{aligned}V_{4i}(k)={}& V_{41}(k)+V_{42}(k) \\ ={}& \sum_{j=-\sigma_{M}}^{-\sigma_{m}-1}\sum _{i=k+j}^{k-1}\mu ^{k-i-1} x^{T}(i)S_{1}x(i)+ \sum_{l=-\sigma_{M}}^{-\sigma_{m}-1}\sum _{j=l}^{-1}\sum_{i=k+j}^{k-1} \mu^{k-i-1} x^{T}(i)S_{2}x(i) \\ & +\sum_{j=-\tau_{(}M)}^{-\tau_{m}-1}\sum _{i=k+j}^{k-1}\rho ^{k-i-1} y^{T}(i)S_{3}y(i)+ \sum_{l=-\tau_{M}}^{-\tau_{m}-1}\sum _{j=l}^{-1}\sum_{i=k+j}^{k-1} \rho^{k-i-1} y^{T}(i)S_{4}y(i),\end{aligned} \\& \begin{aligned}V_{5i}(k) ={}& V_{51}(k)+V_{52}(k) \\ ={}& \hat{\sigma}\sum_{j=-\sigma_{M}}^{-\sigma_{m}-1}\sum _{i=k+j}^{k-1}\mu^{k-i-1} \eta^{T}(i)Z_{1}\eta(i)+\frac{\check {\sigma}}{2}\sum _{l=-\sigma_{M}}^{-\sigma_{m}-1}\sum_{j=l}^{-1} \sum_{i=k+j}^{k-1}\mu^{k-i-1} \eta^{T}(i)Z_{2}\eta(i) \\ & +\hat{\tau}\sum_{j=-\tau_{(}M)}^{-\tau_{m}-1}\sum _{i=k+j}^{k-1}\rho^{k-i-1} \zeta^{T}(i)Z_{3}\zeta(i)+\frac{\check {\tau}}{2}\sum _{l=-\tau_{M}}^{-\tau_{m}-1}\sum_{i=k+j}^{k-1} \rho ^{k-i-1} \zeta^{T}(i)Z_{4}\zeta(i),\end{aligned} \end{aligned}$$

where $\eta(k) = x(k+1)-x(k)$ and $\zeta(k) = y(k+1)-y(k)$. Calculating the forward difference of V(k) by defining $\Delta V(k) = V(k+1)-V(k)$ along the solution of (1) and (3), we obtain

13 $$\begin{aligned}& \Delta V_{a1}(k)-(\mu-1)V_{a1}(k)+\Delta V_{a2}(k)-(\rho -1)V_{a2}(k) \\& \quad= \sum_{i=1}^{5} \bigl[\Delta V_{a1_{i}}(k)-(\mu -1)V_{a1_{i}}(k)+\Delta\bigl(V_{a2_{i}}(k) \bigr)-(\rho-1)V_{a2_{i}}(k) \bigr], \\& \qquad \text{where } a = 1, 2,\ldots,5, \end{aligned}$$

14 $$\begin{aligned}& \Delta V_{11_{i}}(k)-(\mu-1)V_{11_{i}}(k)+\Delta \bigl(V_{12_{i}}(k)\bigr)-(\rho-1)V_{12_{i}}(k) \\& \quad= x^{T}(k+1)P_{1i}x(k+1)-\mu x^{T}P_{1i}x(k)+y^{T}(k+1)P_{2i}y(k+1) \\& \qquad{} -\rho y^{T}P_{2i}y(k) \\& \quad= \bigl[A_{i}(k)x(k-\gamma_{1})+B_{i}(k)f \bigl(y(k)\bigr)+C_{i}f\bigl(y\bigl(k-\tau (k)\bigr)\bigr)+u(k) \bigr]^{T}\bar{P}_{1i} \\& \qquad{} \times \bigl[A_{i}(k)x(k-\gamma _{1})+B_{i}(k)f \bigl(y(k)\bigr)+C_{i}f\bigl(y\bigl(k-\tau(k)\bigr)\bigr)+u(k) \bigr] \\& \qquad{} -\mu x^{T}(k)P_{1i}x(k) \bigl[D_{i}(k)y(k- \gamma _{2})+E_{i}(k)g\bigl(x(k)\bigr)+F_{i}(k) \\& \qquad{} \times g\bigl(x\bigl(k-\sigma(k)\bigr)\bigr)+v(k) \bigr]^{T} \bar{P}_{2i} \bigl[D_{i}(k)y(k-\gamma_{2})+E_{i}(k)g \bigl(x(k)\bigr) \\ & \qquad{} +F_{i}(k)g\bigl(x\bigl(k-\sigma(k)\bigr)\bigr)+v(k) \bigr]- \rho y_{T}(k)P_{2i}y(k), \end{aligned}$$

15 $$\begin{aligned}& \Delta V_{21_{i}}(k)-(\mu-1)V_{21_{i}}(k)+ \Delta V_{22_{i}}(k) (\rho -1)V_{22_{i}}(k) \\ & \quad\leq x^{T}(k)W x(k)-\mu^{\sigma_{M}}x^{T}\bigl(k- \sigma(k)\bigr)W x\bigl(k-\sigma(k)\bigr) \\ & \qquad{} +\sum_{i=k+1-\sigma_{M}}^{k-\sigma_{m}} \mu^{k-i}x^{T}(i)W x(i)+\hat{\sigma}x^{T}(k)W x(k) - \sum_{i=k+1-\sigma_{M}}^{k-\sigma_{m}}\mu^{k-i}x^{T}(i)W x(i) \\ & \qquad{} +y^{T}(k)w_{1}y(k) -\rho^{\tau_{M}}y^{T} \bigl(k-\tau(k)\bigr)W_{1} y\bigl(k-\tau(k)\bigr) \\ & \qquad{} +\sum _{i=k+1-\tau_{M}}^{k-\tau_{m}}\rho^{k-i}y^{T}(i)W_{1} y(i)+\hat{ \tau}y^{T}(k)W_{1} y(k) -\sum_{i=k+1-\tau_{M}}^{k-\tau_{m}} \rho^{k-i}y^{T}(i)W_{1} y(i), \end{aligned}$$

16 $$\begin{aligned}& \Delta V_{31_{i}}(k)-(\mu-1)V_{31_{i}}(k)+\Delta V_{32_{i}}(k)-(\rho -1)V_{32_{i}}(k) \\ & \quad\leq (1+\hat{\sigma})g^{T}\bigl(x(k)\bigr)R g\bigl(x(k)\bigr)- \mu^{\sigma_{M}} g^{T}\bigl(x\bigl(k-\sigma(k)\bigr)\bigr)R g\bigl(k- \sigma(k)\bigr) \\ & \qquad{} +\sum_{i=k+1-\sigma_{M}}^{k-\sigma_{m}}\mu ^{k-i}g^{T}\bigl(x(i)\bigr)R g\bigl(x(i)\bigr) -\sum _{i=k+1-\sigma_{M}}^{k-\sigma_{m}}\mu ^{k-i}g^{T} \bigl(x(i)\bigr)R g\bigl(x(i)\bigr) \\ & \qquad{} +(1+\hat{\tau})f^{T}\bigl(y(k) \bigr)R_{1} f\bigl(y(k)\bigr)-\rho^{\tau _{M}}f^{T}\bigl(y \bigl(k-\tau(k)\bigr)\bigr)R_{1} f\bigl(y\bigl(k-\tau(k)\bigr)\bigr) \\ & \qquad{} +\sum _{i=k+1-\tau_{M}}^{k-\tau_{m}}\rho^{k-i}f^{T} \bigl(y(i)\bigr)R_{1} f\bigl(y(i)\bigr) -\sum_{i=k+1-\tau_{M}}^{k-\tau_{m}}\rho ^{k-i}f^{T}\bigl(y(i)\bigr)R_{1} f\bigl(y(i)\bigr), \end{aligned}$$

17 $$\begin{aligned}& \Delta V_{41_{i}}(k)-(\mu-1)V_{41_{i}}(k)+\Delta V_{42_{i}}(k)-(\rho -1)V_{42_{i}}(k) \\ & \quad\leq (1+\hat{\sigma})x^{T}(k)S_{1}x(k) \\ & \qquad{} -\mu^{\sigma_{m}+1} g^{T}\bigl(x\bigl(k-\sigma(k)\bigr)\bigr)R g\bigl(k-\sigma(k)\bigr)\sum_{i=k-\sigma_{M}}^{k-\sigma_{m}-1} x^{T}(i)S_{1}x(i) \\ & \qquad{} +\frac{\hat{\sigma}}{2}x^{T}(k)S_{2}x(k) -\mu^{\sigma_{m}+1}\sum_{l=-\sigma_{M}}^{-\sigma_{m}-1}\sum _{j=k+l}^{k-1}x^{T}(j)S_{2}x(j)+\hat{\tau}y^{T}(k)S_{3}y(k) \\ & \qquad{}-\rho^{\tau_{m}+1}\sum_{i=k-\tau_{M}}^{k-\tau_{m}-1}y^{T}(i)S_{3} y(i)\frac{\hat{\tau}}{2}y^{T}(k)S_{4}y(k) - \rho^{\tau_{m}+1}\sum_{l=-\sigma_{M}}^{-\sigma_{m}-1}\sum _{j=k+l}^{k-1}y^{T}(j)S_{4}y(j), \end{aligned}$$

18 $$\begin{aligned}& \Delta V_{51_{i}}(k)-(\mu-1)V_{51_{i}}(k)+ \Delta V_{52_{i}}(k)-(\rho-1)V_{52_{i}}(k) \\ & \quad\leq \hat{\sigma}^{2}\eta^{T}(k)Z_{1}\eta(k)- \hat{\sigma}\mu ^{\sigma_{m}+1}\sum_{i=k-\sigma_{M}}^{k-\sigma_{m}-1} \eta ^{T}(i)Z_{1}\eta(i)\frac{\check{\sigma}^{2}}{4}\eta ^{T}(k)Z_{2}\eta(k) \\ & \qquad{} -\frac{\check{\sigma}}{2}\mu^{\sigma_{m}+1}\sum_{l=-\sigma_{M}}^{-\sigma_{m}-1} \sum_{j=k+l}^{k-1}\eta ^{T}(j)Z_{2} \eta(j) +\hat{\tau}^{2}\zeta^{T}(k)Z_{3}\zeta(k) \\ & \qquad{} -\hat{\tau}\rho^{\tau_{m}+1}\sum _{j=k-\tau_{M}}^{k-\sigma _{m}-1}\zeta^{i}Z_{3} \zeta(i)\frac{\check{\tau}^{2}}{4}\zeta ^{T}(k)Z_{3} \zeta(k) - \frac{\check{\tau}}{2}\rho^{\tau _{m}+1}\sum_{l=-\sigma_{M}}^{-\sigma_{m}-1}\sum _{j=k+l}^{k-1}\zeta^{T}(j)Z_{4} \zeta(j) \\& \quad\leq \bigl[\bigl(A(k)-I\bigr)x(k-\gamma_{1})+B(k)f\bigl(y(k) \bigr)+c(k)f\bigl(y\bigl(k-\tau (k)\bigr)\bigr)+v(k) \bigr] \\ & \qquad{} -\mu^{\sigma_{m}+1}\hat{\sigma}\sum _{i=k-\sigma_{M}}^{k-\sigma_{m}-1}\eta^{T}(i)Z_{1} \eta(i)-\mu^{\sigma_{m}+1}\frac{\check{\sigma}}{2}\sum_{l=-\sigma_{M}}^{j=-\sigma_{m}-1}\sum _{j=k+l}^{k-1}\eta^{T}(j)Z_{2} \eta(j) \\ & \qquad{} +\bigl[E(k)g\bigl(x(k)\bigr)+F(k)g\bigl(y\bigl(k-\sigma (k)\bigr)\bigr)+u(k) \bigr] \\ & \qquad{} -\rho^{\tau_{m}}\hat{\tau}\sum _{i=k-\tau _{M}}^{k-\sigma_{m}-1}\zeta^{T}(i)Z_{3} \zeta(i) \rho^{\tau_{m}+1}\frac{\check{\tau}}{2}\sum_{l=-\sigma _{M}}^{j=-\sigma_{m}-1} \sum_{j=k+l}^{k-1}\zeta^{T}(j)Z_{4} \zeta (j). \end{aligned}$$

Using Lemma 2.5(i), the first summation term in $\Delta V_{5i}$ can be written as

$$\begin{aligned} -\hat{\sigma}\sum_{s=k-\sigma_{M}}^{k-\sigma_{m}-1}\eta ^{T}(s)Z_{1}\eta(s)={}&-\hat{\sigma}\sum _{s=k-\sigma_{M}}^{k-\sigma (k)-1}\eta^{T}(s)Z_{1} \eta(s)-\hat{\sigma}\sum_{s=k-\sigma (k)}^{k-\sigma_{m}-1} \eta^{T}(s)Z_{1}\eta(s) \\ \leq{}&\frac{-\hat{\sigma}}{\sigma_{1}}\sum_{s=k-\sigma _{M}}^{k-\sigma(k)-1} \eta^{T}(s)Z_{1}\sum_{s=k-\sigma _{M}}^{k-\sigma(k)-1} \eta(s)\\ & -\frac{\hat{\sigma}}{\sigma_{2}}\sum_{s=k-d(k)}^{k-\sigma (m)-1} \eta^{T}(s)Z_{1}\sum_{s=k-\sigma(k)}^{k-\sigma_{m}-1} \eta (s) \\ \leq{}&\frac{-\hat{\sigma}}{\sigma_{1}} \bigl[x\bigl(k-\sigma (k)\bigr)-x(k- \sigma_{M}) \bigr]^{T}Z_{1} \bigl[x\bigl(k- \sigma(k)\bigr)-x(k-\sigma _{M}) \bigr] \\ & -\frac{\hat{\sigma}}{\sigma_{2}} \bigl[x(k-\sigma _{m})-x\bigl(k-\sigma(k) \bigr) \bigr]^{T}Z_{1} \bigl[x(k-\sigma_{m})-x \bigl(k-\sigma (k)\bigr) \bigr].\end{aligned} $$

Similarly,

19 $$\begin{aligned}[b] &{-}\hat{\tau}\sum_{s=k-\tau_{M}}^{k-\tau_{m}-1}\zeta ^{T}(s)Z_{3}\zeta(s) \\ &\quad\leq \frac{-\hat{\tau}}{\tau_{1}} \bigl[y \bigl(k-\tau(k)\bigr)-y(k-\tau _{M}) \bigr]^{T}Z_{3} \bigl[y\bigl(k-\tau(k)\bigr)-y(k-\tau_{M}) \bigr] \\ &\qquad{} -\frac{\hat{\tau}}{\tau_{2}} \bigl[y(k-\tau_{m})-y\bigl(k-\tau (k) \bigr) \bigr]^{T}Z_{3} \bigl[y(k-\tau_{m})-y \bigl(k-\tau(k)\bigr) \bigr]. \end{aligned} $$

Further, using Lemma 2.5(ii), the second summation term in $\Delta V_{5i}(k)$ becomes

20 $$\begin{aligned} \frac{\check{\sigma}}{2}\sum_{l=-\sigma_{M}}^{-\sigma_{m}-1}\sum _{j=k+l}^{k-1}\eta^{T}(j)Z_{2} \eta(j) =&\frac{\check{\sigma }}{2}\sum_{l=-\sigma_{M}}^{-\sigma(k)-1} \sum_{j=k+l}^{k-1}\eta ^{T}(j)Z_{2} \eta(j) +\frac{\check{\sigma}}{2}\sum_{l=-\sigma(k)}^{-\sigma_{m}-1} \sum_{j=k+l}^{k-1}\eta^{T}(j)Z_{2} \eta(j) \\ \geq&\frac{\check{\sigma}}{\sigma_{3}} \Biggl[\sum_{l=-\sigma _{M}}^{-\sigma(k)-1} \sum_{j=k+l}^{k-1}\eta^{T}(j)Z_{2} \sum_{l=-\sigma(k)}^{-\sigma_{m}-1}\sum _{j=k+l}^{k-1}\eta(j) \Biggr] \\ & {} +\frac{\check{\sigma}}{\sigma{4}} \Biggl[\sum_{l=-\sigma (k)}^{-\sigma_{m}-1}\sum _{j=k+l}^{k-1}\eta^{T}(j)Z_{2}\sum_{l=-\sigma(k)}^{-\sigma_{m}-1} \sum_{j=k+l}^{k-1}\eta(j) \Biggr] \\ \geq&\frac{\check{\sigma}}{\sigma_{3}} \Biggl[\sigma_{1}x(k)-\sum _{i=k-\sigma_{M}}^{k-\sigma(k)-1}x(i) \Biggr]^{T}Z_{2} \Biggl[\sigma _{1}x(k)-\sum_{i=k-\sigma_{M}}^{k-\sigma_{m}-1}x(i) \Biggr] \\ & {}+\frac{\check{\sigma}}{\sigma_{4}} \Biggl[\sigma_{2}x(k)-\sum_{i=k-\sigma(k)}^{k-\sigma_{m}-1}x(i) \Biggr]^{T}Z_{2} \Biggl[\sigma_{2}x(k)-\sum _{i=k-\sigma(k)}^{k-\sigma _{m}-1}x(i) \Biggr]. \end{aligned}$$

By reciprocally convex combination Lemma 2.6, if LMIs in (8) hold, then the following inequalities hold:

$$\begin{gathered} \begin{pmatrix} \sqrt{\frac{\alpha_{1}}{\alpha_{2}}}(x^{T}(k-\sigma (k))-x^{T}(k-\sigma_{M}))\\ -\sqrt{\frac{\alpha_{2}}{\alpha_{1}}}(x^{T}(k-\sigma _{M})-x^{T}(k-\sigma(k))) \end{pmatrix} ^{T} \begin{pmatrix} Z_{1} &U_{1}\\ * &Z_{1} \end{pmatrix}\\ \quad\times{} \begin{pmatrix} \sqrt{\frac{\alpha_{1}}{\alpha_{2}}}(x^{T}(k-\sigma (k))-x^{T}(k-\sigma_{M}))\\ -\sqrt{\frac{\alpha_{2}}{\alpha_{1}}}(x^{T}(k-\sigma _{M})-x^{T}(k-\sigma(k))) \end{pmatrix} \geq 0, \\ \begin{pmatrix} \sqrt{\frac{\beta_{1}}{\beta_{2}}}(\sigma_{1}x^{T}(k)-\sum_{i=k-\sigma_{M}}^{k-\sigma(k)-1}x^{T}(i))\\ -\sqrt{\frac{\beta_{2}}{\beta_{1}}}(\sigma_{2}x^{T}(k)-\sum_{i=k-\sigma(k)}^{k-\sigma_{m}-1}x^{T}(i)) \end{pmatrix} ^{T} \begin{pmatrix} Z_{2} &U_{2}\\ * &Z_{2} \end{pmatrix}\\ \quad\times{} \begin{pmatrix} \sqrt{\frac{\beta_{1}}{\beta_{2}}}(\sigma_{1}x^{T}(k)-\sum_{i=k-\sigma_{M}}^{k-\sigma(k)-1}x^{T}(i))\\ -\sqrt{\frac{\beta_{2}}{\beta_{1}}}(\sigma_{2}x^{T}(k)-\sum_{i=k-\sigma(k)}^{k-\sigma_{m}-1}x^{T}(i)) \end{pmatrix} \geq 0,\end{gathered} $$

where $\alpha_{1}=\frac{\sigma_{1}}{\hat{\sigma}}$, $\alpha _{2}=\frac{\sigma_{2}}{\hat{\sigma}}$, $\beta_{1}=\frac{\sigma _{3}}{\check{\sigma}}$, $\beta_{1}=\frac{\sigma_{4}}{\check {\sigma}}$ with $\sigma_{1}=(\sigma_{M}-\sigma(k))$, $\sigma_{2}=(\sigma(k)-\sigma_{m})$, $\sigma_{3}=(\sigma_{M}-\sigma(k))(\sigma_{M}+\sigma(k)+1)$, $\sigma_{4}=(\sigma(k)-\sigma_{m})(\sigma(k)+\sigma_{m}+1)$.

Similarly,

$$\begin{aligned} \frac{\check{\tau}}{2}\sum_{l=-\tau_{M}}^{-\tau_{m}-1}\sum _{j=k+l}^{k-1}\zeta^{T}(j)Z_{4} \zeta(j)={}&\frac{\check{\tau }}{2}\sum_{l=-\tau_{M}}^{-\tau(k)-1} \sum_{j=k+l}^{k-1}\zeta ^{T}(j)Z_{4} \zeta(j) +\frac{\check{\tau}}{2}\sum_{l=-\tau(k)}^{-\tau_{m}-1} \sum_{j=k+l}^{k-1}\zeta^{T}(j)Z_{4} \zeta(j) \\ \geq{}&\frac{\check{\tau}}{\tau_{3}} \Biggl[\sum_{l=-\tau _{M}}^{-\tau(k)-1} \sum_{j=k+l}^{k-1}\zeta^{T}(j)Z_{4} \sum_{l=-\tau (k)}^{-\tau_{m}-1}\sum _{j=k+l}^{k-1}\zeta(j) \Biggr] \\ & +\frac{\check{\tau}}{\tau{4}} \Biggl[\sum_{l=-\tau(k)}^{-\tau _{m}-1}\sum _{j=k+l}^{k-1}\zeta^{T}(j) Z_{4}\sum_{l=-\tau(k)}^{-\tau_{m}-1} \sum_{j=k+l}^{k-1}\zeta(j) \Biggr] \\ \geq{}&\frac{\check{\tau}}{\tau_{3}} \Biggl[\tau_{1}y(k)-\sum _{i=k-\tau_{M}}^{k-\tau(k)-1}y(i) \Biggr]^{T}Z_{4} \Biggl[\tau _{1}y(k)-\sum_{i=k-\tau_{M}}^{k-\tau_{m}-1}y(i) \Biggr] \\ & +\frac{\check{\tau}}{\tau_{4}} \Biggl[\tau_{2}y(k)-\sum_{i=k-\tau(k)}^{k-\tau_{m}-1}y(i) \Biggr]^{T}Z_{2} \Biggl[\tau_{2}y(k)-\sum _{i=k-\tau(k)}^{k-\tau_{m}-1}y(i) \Biggr].\end{aligned} $$

By reciprocally convex combination Lemma 2.6, if LMIs in (9) hold, then the following inequalities hold:

$$\begin{aligned}& \begin{gathered}\begin{pmatrix} \sqrt{\frac{\alpha_{3}}{\alpha_{4}}}(y^{T}(k-\tau(k))-y^{T}(k-\tau _{M}))\\ -\sqrt{\frac{\alpha_{4}}{\alpha_{3}}}(y^{T}(k-\tau _{M})-y^{T}(k-\tau(k))) \end{pmatrix} ^{T} \begin{pmatrix} Z_{3} &U_{3}\\ * &Z_{3} \end{pmatrix}\\ \quad\times{} \begin{pmatrix} \sqrt{\frac{\alpha_{3}}{\alpha_{4}}}(y^{T}(k-\tau(k))-y^{T}(k-\tau _{M}))\\ -\sqrt{\frac{\alpha_{4}}{\alpha_{3}}}(y^{T}(k-\tau _{M})-y^{T}(k-\tau(k))) \end{pmatrix} \geq 0,\end{gathered} \\& \begin{gathered}\begin{pmatrix} \sqrt{\frac{\beta_{3}}{\beta_{4}}}(\tau_{1}y^{T}(k)-\sum_{i=k-\sigma_{M}}^{k-\sigma(k)-1}y^{T}(i))\\ -\sqrt{\frac{\beta_{4}}{\beta_{3}}}(\tau_{2}y^{T}(k)-\sum_{i=k-\sigma(k)}^{k-\sigma_{m}-1}y^{T}(i)) \end{pmatrix} ^{T} \begin{pmatrix} Z_{4} &U_{4}\\ * &Z_{4} \end{pmatrix}\\\quad\times{} \begin{pmatrix} \sqrt{\frac{\beta_{3}}{\beta_{4}}}(\tau_{1}y^{T}(k)-\sum_{i=k-\sigma_{M}}^{k-\sigma(k)-1}y^{T}(i))\\ -\sqrt{\frac{\beta_{4}}{\beta_{3}}}(\tau_{2}y^{T}(k)-\sum_{i=k-\sigma(k)}^{k-\sigma_{m}-1}y^{T}(i)) \end{pmatrix} \geq 0,\end{gathered} \end{aligned}$$

where $\alpha_{3}=\frac{\tau_{1}}{\hat{\tau}}$, $\alpha _{4}=\frac{\tau_{2}}{\hat{\tau}}$, $\beta_{1}=\frac{\tau _{3}}{\check{\tau}}$, $\beta_{1}=\frac{\tau_{4}}{\check{\tau }}$ with $\tau_{1}=(\tau_{M}-\tau(k))$, $\tau_{2}=(\tau(k)-\tau_{m})$, $\tau_{3}=(\tau_{M}-\tau(k))(\tau _{M}+\tau(k)+1)$, $\tau_{4}=(\tau(k)-\tau_{m})(\tau(k)+\tau_{m}+1)$.

Then inequalities (19) and (20) can be rewritten as

21 $$\begin{aligned}& \begin{aligned}[b] -\hat{\sigma}\sum_{s=k-\sigma_{M}}^{k-\sigma_{m}-1} \eta ^{T}(s)Z_{1}\eta(s) \leq{}& {-} \begin{pmatrix} x^{T}(k-\sigma(k))-x^{T}(k-\sigma_{M}))\\ x^{T}(k-\sigma_{m})-x^{T}(k-\sigma(k)) \end{pmatrix} ^{T} \begin{pmatrix} Z_{1} &U_{1}\\ * &Z_{1} \end{pmatrix} \\ &\times \begin{pmatrix} x(k-\sigma(k))-x(k-\sigma_{M})\\ x(k-\sigma_{m})-x(k-\sigma(k)) \end{pmatrix}, \end{aligned} \end{aligned}$$

22 $$\begin{aligned}& \begin{aligned}[b]-\frac{\check{\sigma}}{2}\sum_{l=-\sigma_{M}}^{-\sigma_{m}-1}\sum _{j=k+l}^{k-1} \eta^{T}(j)Z_{2} \eta(j) \leq{}& {-} \begin{pmatrix} \sigma_{1}x^{T}(k)-\sum_{i=k-\sigma_{M}}^{k-\sigma(k)-1}x^{T}(i)\\ \sigma_{2}x^{T}(k)-\sum_{i=k-\sigma(k)}^{k-\sigma_{m}-1}x^{T}(i) \end{pmatrix} ^{T} \begin{pmatrix} Z_{2} &U_{2}\\ * &Z_{2} \end{pmatrix} \\ &\times \begin{pmatrix} \sigma_{1}x^{T}(k)-\sum_{i=k-\sigma_{M}}^{k-\sigma(k)-1}x^{T}(i)\\ \sigma_{2}x^{T}(k)-\sum_{i=k-\sigma(k)}^{k-\sigma_{m}-1}x^{T}(i) \end{pmatrix} .\end{aligned} \end{aligned}$$

Similarly,

23 $$\begin{aligned}& \begin{aligned}[b] -\hat{\tau}\sum_{s=k-\sigma_{M}}^{k-\sigma_{m}-1}\zeta ^{T}(s)Z_{3}\zeta(s) \leq{}&{ -} \begin{pmatrix} y^{T}(k-\tau(k))-y^{T}(k-\tau_{M})\\ y^{T}(k-\tau_{m})-y^{T}(k-\tau(k)) \end{pmatrix} ^{T} \begin{pmatrix} Z_{3} &U_{3}\\ * &Z_{3} \end{pmatrix} \\ &\times \begin{pmatrix} y(k-\tau(k))-y(k-\tau_{M})\\ y(k-\tau_{m})-y(k-\tau(k)) \end{pmatrix},\end{aligned} \end{aligned}$$

24 $$\begin{aligned}& \begin{aligned}[b]-\frac{\check{\tau}}{2}\sum_{l=-\tau_{M}}^{-\tau_{m}-1}\zeta ^{T}(j)Z_{4}\zeta(j) \leq{}& \begin{pmatrix} \tau_{1}y^{T}(k)-\sum_{i=k-\tau_{M}}^{k-\tau(k)-1}y^{T}(i)\\ \tau_{2}y^{T}(k)-\sum_{i=k-\tau(k)}^{k-\tau_{m}-1}y^{T}(i) \end{pmatrix} ^{T} \begin{pmatrix} Z_{4} &U_{4}\\ * &Z_{4} \end{pmatrix} \\ &\times \begin{pmatrix} \tau_{1}y^{T}(k)-\sum_{i=k-\tau_{M}}^{k-\tau(k)-1}y^{T}(i)\\ \tau_{2}y^{T}(k)-\sum_{i=k-\tau(k)}^{k-\tau_{m}-1}y^{T}(i) \end{pmatrix} .\end{aligned} \end{aligned}$$

It is noted that when $\sigma(k)=\sigma_{m}$ or $\sigma(k)=\sigma _{M}$ and $\tau(k)=\tau_{m}$ or $\tau(k)=\tau_{M}$, we have $x(k-\sigma(k))-x(k-\sigma_{M})=0$ or $x(k-\sigma_{m})-x(k-\sigma(k))=0$ and $y(k-\tau(k)) -y(k-\tau_{M})=0$ or $y(k-\tau_{m})-y(k-\tau(k))=0$, respectively. So, inequalities (20) and (21) still hold.

For any matrices $u_{5}$, $u_{6}$, $u_{7}$, $u_{8}$, the following equalities hold:

25 $$\begin{aligned}& \mu^{\sigma_{m}+1} \Biggl[x^{T}(k-\sigma_{m})U_{5}x(k- \sigma _{m})-x^{T}(k-\sigma_{M})U_{5}x(k- \sigma_{M}) \\& \quad-\sum_{s=k-\sigma_{M}}^{k-\sigma_{m}-1} \bigl[ \eta^{T}(s)U_{5}\eta (s)+2x^{T}(s)U_{5} \eta(s) \bigr] \Biggr]=0, \end{aligned}$$

26 $$\begin{aligned}& \begin{aligned}[b]&\mu^{\sigma_{m}+1} \Biggl[\hat{\sigma}x^{T}(k)U_{6}x(k- \sigma _{m})-x^{T}(k-\sigma_{M})U_{5}x(k- \sigma_{M}) \\ &\quad-\sum_{s=k-\sigma_{M}}^{k-\sigma_{m}-1} \bigl[ \eta^{T}(j)U_{6}\eta (j)+2x^{T}(j)U_{6} \eta(j) \bigr] \Biggr]=0 ,\end{aligned} \end{aligned}$$

27 $$\begin{aligned}& \rho^{\sigma_{m}+1} \Biggl[y^{T}(k-\sigma_{m})U_{7}y(k- \tau _{m})-y^{T}(k-\tau_{M})U_{7}y(k- \tau_{M}) \\& \quad-\sum_{s=k-\sigma_{M}}^{k-\sigma_{m}-1} \bigl[ \zeta^{T}(s)U_{7}\zeta (s)+2y^{T}(s)U_{7} \zeta(s) \bigr] \Biggr]=0 , \end{aligned}$$

28 $$\begin{aligned}& \rho^{\sigma_{m}+1} \Biggl[\hat{\tau}y^{T}(k)U_{8}y(k)- \sum_{s=k-\sigma_{M}}^{k-\sigma_{m}-1}y^{T}(s)U_{8}x(s) \\& \quad-\sum_{l=-\tau_{M}}^{-\sigma_{m}-1}\sum _{j=k+l}^{k-1} \bigl[\zeta ^{T}(j)U_{8} \zeta(j)+2y^{T}(j)U_{8}\zeta(j) \bigr] \Biggr]=0. \end{aligned}$$

On the other hand, from Assumption I, we have

$$\begin{gathered} \bigl(f_{i}\bigl(x_{i}(k)\bigr)-F_{i}^{-} \bigl(x_{i}(k)\bigr) \bigr)- \bigl(f_{i} \bigl(x_{i}(k)\bigr)-F_{i}^{+} \bigl(x_{i}(k)\bigr) \bigr)\leq0, \\ \bigl(g_{i}\bigl(y_{i}(k)\bigr)-G_{i}^{-} \bigl(y_{i}(k)\bigr) \bigr)- \bigl(g_{i} \bigl(y_{i}(k)\bigr)-G_{i}^{+} \bigl(y_{i}(k)\bigr) \bigr)\leq0,\end{gathered} $$

which is equivalent to

$$\begin{aligned} \begin{pmatrix} x(k)\\ g(x(k)) \end{pmatrix} ^{T} \begin{pmatrix} G_{i}^{-}G_{i}^{+}e_{i}e_{i}^{T} &-\frac {-G_{i}^{-}+G_{i}^{+}}{2}e_{i}e^{T}\\ \frac{G_{i}^{-}+G_{i}^{+}}{2}e_{i}e^{T} &e_{i}e^{T} \end{pmatrix} \begin{pmatrix} x(k)\\ g(x(k)) \end{pmatrix} _{i=1,2,\ldots,k} \leq0 \end{aligned}$$

and

$$\begin{aligned} \begin{pmatrix} y(k)\\ f(y(k)) \end{pmatrix} ^{T} \begin{pmatrix} F_{i}^{-}F_{i}^{+}e_{i}e_{i}^{T} &-\frac {-F_{i}^{-}+F_{i}^{+}}{2}e_{i}e^{T}\\ \frac{F_{i}^{-}+F_{i}^{+}}{2}e_{i}e^{T} &e_{i}e^{T} \end{pmatrix} \begin{pmatrix} y(k)\\ f(y(k)) \end{pmatrix} _{i=1,2,\ldots,n} \leq0, \end{aligned}$$

where $e_{i}$ denotes the unit column vector having the element 1 on its *r*th row and zero elsewhere. Let $H_{1} = \operatorname{diag}\{ h_{11},h_{12},\ldots,h_{1n}\}$, $H_{2} = \operatorname{diag}\{h_{21},h_{22},\ldots,h_{2n}\}$, $H_{3} = \operatorname{diag}\{h_{31},h_{32},\ldots, h_{3n}\}$, $H_{4} = \operatorname{diag}\{ h_{41},h_{42},\ldots,h_{4n}\}$.

Then

29 $$\begin{gathered} \sum_{i=0}^{n}h_{1i} \begin{pmatrix} x(k)\\ g(x(k)) \end{pmatrix} ^{T} \begin{pmatrix} G_{i}^{-}G_{i}^{+}e_{i}e_{i}^{T} &-\frac {-G_{i}^{-}+G_{i}^{+}}{2}e_{i}e^{T}\\ -\frac{G_{i}^{-}+G_{i}^{+}}{2}e_{i}e^{T} &e_{i}e^{T} \end{pmatrix} \begin{pmatrix} x(k)\\ g(x(k)) \end{pmatrix} \leq 0, \\ \begin{pmatrix} x(k)\\ g(x(k)) \end{pmatrix} ^{T} \begin{pmatrix} G_{1}H_{1} &-G_{2}H_{1}\\ G_{1}H_{1} &H_{1} \end{pmatrix} \begin{pmatrix} x(k)\\ g(x(k)) \end{pmatrix} \leq 0.\end{gathered} $$

Similarly,

30 $$ \begin{pmatrix} y(k)\\ f(y(k)) \end{pmatrix} ^{T} \begin{pmatrix} F_{3}H_{3} &-F_{4}H_{3}\\ G_{4}H_{3} &H_{3} \end{pmatrix} \begin{pmatrix} y(k)\\ f(y(k)) \end{pmatrix} \leq 0. $$

Similarly, one can get

$$ \begin{pmatrix} x(k-\sigma(k))\\ g(x(k-\sigma(k))) \end{pmatrix} ^{T} \begin{pmatrix} G_{1}H_{2} &-G_{2}H_{2}\\ G_{2}H_{2} &H_{2} \end{pmatrix} \begin{pmatrix} x(k-\sigma(k))\\ g(x(k-\sigma(k))) \end{pmatrix} \leq 0 $$

and

$$ \begin{pmatrix} y(k-\tau(k))\\ f(y(k-\tau(k))) \end{pmatrix} ^{T} \begin{pmatrix} G_{3}H_{4} &-G_{4}H_{4}\\ -G_{4}H_{4} &H_{4} \end{pmatrix} \begin{pmatrix} y(k-\tau(k))\\ f(y(k-\tau(k))) \end{pmatrix} \leq 0. $$

Then from (13) and adding (25)-(30) gives

31 $$\begin{aligned}[b] &\Delta V_{a1}(k)-(\mu-1)V_{a1}(k)-V^{T}(k)Q V(k)+\Delta V_{a2}(k)-(\rho-1)V_{a2}(k)-u^{T}(k)Q_{1} u(k) \\ &\quad\leq \varpi^{T}(k) \biggl[\Psi+\Psi_{1}^{T}P_{1i} \Psi_{1}+\hat{\sigma }^{2}\Psi_{2}^{T}Z_{1} \Psi_{2}+\frac{\check{\sigma}^{2}}{4}\Psi _{2}^{T}Z_{2} \Psi_{2} \biggr]\varpi(k) \\ &\qquad{} -\mu^{\sigma_{m}+1} \Biggl[\sum_{s=k-\sigma_{M}}^{k-\sigma _{m}-1} \varpi_{1}^{T}(s)\Psi_{3}\varpi_{1}^{T}(s) -\sum_{l=-\sigma_{M}}^{-\sigma_{m}-1}\sum _{j=k+l}^{k-1}\varpi_{1}^{T}(j)\Psi_{4} \varpi_{1}^{T}(j) \Biggr] \\ &\quad\quad{} +\Xi^{T}(k) \biggl[\Phi+ \Phi_{1}^{T}P_{2i}\Phi_{1}+\hat{\tau }^{2}\Phi_{2}^{T}Z_{3} \Phi_{2}+\frac{\check{\tau}^{2}}{4}\Phi_{2}^{T}Z_{4} \Phi_{2} \biggr]\Xi(k) \\ &\qquad{} -\rho^{\tau_{m}+1} \Biggl[\sum _{s=k-\tau_{M}}^{k-\tau_{m}-1}\Xi _{1}^{T}(s) \Phi_{3}\Xi_{1}^{T}(s) -\sum_{l=-\sigma_{M}}^{-\sigma_{m}-1}\sum _{j=k+l}^{k-1}\Xi _{1}^{T}(j) \Phi_{4}\Xi_{1}^{T}(j) \Biggr],\end{aligned} $$

where

$$\begin{aligned}& \Psi= \begin{pmatrix} \psi{\Pi}_{1,1,i}& 0& 0& 0& 0& 0& 0& 0& 0& 0\\ *& \Psi_{2,2,i}& 0& 0& 0& G_{2}H_{1}& 0& \Psi_{12}& \Psi_{13}& 0\\ *& *& \Psi_{3,3,i}& \Psi_{3,4,i}& \mu^{\sigma_{m}+1}U_{1}^{T}& 0& 0& 0& 0& 0\\ *& *& *& \Psi_{4,4,i}& \Psi_{4,5,i}& 0& G_{2}H_{2}& 0& 0& 0\\ *& *& *& *& \Psi_{5,5,i}& 0& 0& 0& 0& 0\\ *& *& *& *& *& \Psi_{6,6,i}& 0& 0& 0& 0\\ *& *& *& *& *& *& \Psi_{7,7,i}& 0& 0& 0\\ *& *& *& *& *& *& *& \Psi_{8,8,i}& \Psi_{8,9,i}& 0\\ *& *& *& *& *& *& *& *& \Psi_{9,9,i}& 0\\ *& *& *& *& *& *& *& *& *& -Q \end{pmatrix}, \\& \Phi= \begin{pmatrix} \phi{\Pi}_{1,1,i}& 0& 0& 0& 0& 0& 0& 0& 0& 0\\ *& \Phi_{2,2,i}& 0& 0& 0& F_{4}H_{3}& 0& \Phi_{32}& \Phi_{33}& 0\\ *& *& \Phi_{3,3,i}& \Phi_{3,4,i}& \rho^{\tau_{m}+1}U_{3}^{T}& 0& 0& 0& 0& 0\\ *& *& *& \Phi_{4,4,i}& \Phi_{4,5,i}& 0& G_{4}H_{4}& 0& 0& 0\\ *& *& *& *& \Phi_{5,5,i}& 0& 0& 0& 0& 0\\ *& *& *& *& *& \Phi_{6,6,i}& 0& 0& 0& 0\\ *& *& *& *& *& *& \Phi_{7,7,i}& 0& 0& 0\\ *& *& *& *& *& *& *& \Phi_{8,8,i}& \Phi_{8,9,i}& 0\\ *& *& *& *& *& *& *& *& \Phi_{9,9,i}& 0\\ *& *& *& *& *& *& *& *& *& -Q_{1} \end{pmatrix}, \\& \begin{aligned}\Psi_{2,2,i} ={}& (1+\hat{\sigma})W+\hat{\sigma}\bigl(S_{1}+ \mu^{\sigma _{m}+1}U_{6}\bigr)+\frac{\hat{\sigma}}{2}S_{2}\\&- \mu^{\sigma _{m}+1}\bigl(\sigma_{1}^{2}Z_{2}- \sigma_{2}^{2}Z_{2}-\sigma_{1} \sigma_{2} \bigl(U_{2}+U_{2}^{2}\bigr) \bigr)-\mu P_{1i}-G_{1}H_{1},\end{aligned} \\& \Psi_{12} = \mu^{\sigma_{m}+1} (\sigma_{1}Z_{2}+ \sigma _{2}U_{2}), \qquad \Psi_{13} = \mu^{\sigma_{m}+1}\bigl(\sigma_{2}Z_{2}+\sigma _{1}U_{2}^{T}\bigr), \\& \Psi_{3,3,i} = \mu^{\sigma_{m}+1}(U_{5}-Z_{1}), \qquad \Psi_{3,4,i} = \mu^{\sigma_{m}+1}\bigl(Z_{1}-U_{1}^{T} \bigr), \\& \Psi_{4,4,i} = \mu^{\sigma_{m}+1}\operatorname{sym}(U_{1}-Z_{1})- \mu^{\sigma _{m}}W-G_{1}H_{2}, \\& \Psi_{4,5,i} = \mu^{\sigma_{m}+1}\bigl(Z_{1}-U_{1}^{T} \bigr), \qquad\Psi_{5,5,i} = -\mu^{\sigma_{m}+1}(Z_{1}+U_{5}),\qquad \Psi_{6,6,i} = (1+\hat{\sigma})R-H_{1}, \\& \Psi_{7,7,i} = -\mu^{\sigma_{M}}R-H_{2},\qquad \Psi_{8,8,i} = -\mu^{\sigma_{m}+1}\biggl(Z_{2}+ \frac{1}{\hat{\sigma}}U_{6}\biggr), \\& \Psi_{8,9,i} = -\mu^{\sigma_{m}+1}\biggl(U_{2}^{T}- \frac{1}{\hat {\sigma}}U_{6}\biggr), \qquad\Psi_{9,9,i} = - \mu^{\sigma_{m}+1}\biggl(Z_{2}+\frac{1}{\hat{\sigma }}U_{6} \biggr), \\& \begin{aligned}\Phi_{2,2,i} ={}& (1+\hat{\tau})W_{1}+\hat{\tau} \bigl(S_{3}+\rho^{\tau _{m}+1}U_{8}\bigr)+ \frac{\hat{\tau}}{2}S_{4}\\&-\rho^{\tau_{m}+1}\bigl(\tau _{1}^{2}Z_{4}-\tau_{2}^{2}Z_{4}- \tau_{1}\tau_{2}\bigr)-\rho \bigl(U_{4}+U_{4}^{2} \bigr)P_{2i}-F_{3}H_{3},\end{aligned} \\& \Phi_{32} = \rho^{\tau_{m}+1}(\tau_{1}Z_{4}+ \tau_{2}U_{4}) , \qquad \Phi_{33} = \rho^{\tau_{m}+1}\bigl(\tau_{2}Z_{4}+\tau_{1}U_{4}^{T} \bigr), \\& \Phi_{3,3,i} = \rho^{\tau_{m}+1}(U_{7}-Z_{3}), \qquad \Phi_{3,4,i} = \rho^{\tau_{m}+1}\bigl(Z_{3}-U_{3}^{T} \bigr),\\& \Phi_{4,4,i} = \rho^{\tau_{m}+1}\operatorname{sym}(U_{3}-Z_{3})- \rho^{\tau _{m}}W_{1}-G_{3}H_{4}, \\& \Phi_{4,5,i} = \rho^{\tau_{m}+1}\bigl(Z_{3}-U_{3}^{T} \bigr), \qquad\Phi_{5,5,i} = -\rho^{\tau_{m}+1}(Z_{3}+U_{7}),\qquad \Phi_{6,6,i} = (1+\hat{\tau})R_{1}-H_{3}, \\& \Phi_{7,7,i} = -\rho^{\tau_{M}}R_{1}-H_{4},\qquad \Phi_{8,8,i} = -\rho^{\tau_{m}+1}\biggl(Z_{4}+ \frac{1}{\hat{\tau }}U_{8}\biggr), \\& \Phi_{8,9,i} = -\rho^{\tau_{m}+1}\biggl(U_{4}^{T}- \frac{1}{\hat{\tau }}U_{8}\biggr), \qquad \Phi_{9,9,i} = - \rho^{\tau_{m}+1}\biggl(Z_{4}+\frac{1}{\hat{\tau }}U_{8} \biggr), \\& \Psi_{1} = \bigl[\begin{matrix}A(k)& 0 & 0 & 0 & 0 & B(k) & C(k)& 0 & 0 & I \end{matrix}\bigr], \\& \Psi_{2} = \bigl[\textstyle\begin{array}{c@{\quad}c@{\quad}c@{\quad}c@{\quad}c@{\quad}c@{\quad}c@{\quad}c@{\quad}c@{\quad}c}A(k)-I & 0 & 0 & 0 0 & 0 & B(k) & C(k) & 0 & 0 & I \end{array}\displaystyle \bigr], \\& \Phi_{1} = \bigl[\begin{matrix}D(k) & 0 &0 & 0 & 0 & E(k)& F(k)& 0 & 0 & I \end{matrix}\bigr], \\& \Phi_{2} = \bigl[\begin{matrix}D(k)-I & 0 & 0 & 0& 0 & E(k) & F(k) & 0& 0 & I \end{matrix}\bigr], \\& \begin{aligned}\varpi={}& \Biggl[x^{T}(k-\gamma_{1}) \; x^{T}(k)\; x^{T}(k-\sigma _{m}) \; x^{T}\bigl(k- \sigma(k)\bigr) \; x^{T}(k-\sigma_{M}) \; g^{T} \bigl(x(k)\bigr) \; g^{T}\bigl(x\bigl(k-\sigma(k)\bigr)\bigr) \\ &\times\sum_{i=k-\sigma _{M}}^{k-\sigma(k)-1}x^{T}(i)\; \sum_{i=k-\sigma(k)}^{k-\sigma _{m}-1}x^{T}(i) v^{T}(k) \Biggr]^{T},\end{aligned} \\& \begin{aligned}\Xi={}& \Biggl[y^{T}(k-\gamma_{2}) \; y^{T}(k)\; y^{T}(k-\tau_{m}) \; y^{T}\bigl(k-\tau(k) \bigr) \; y^{T}(k-\tau_{M}) \; f^{T}\bigl(y(k) \bigr)\; f^{T}\bigl(y\bigl(k-\tau(k)\bigr)\bigr) \\ &\times\sum_{i=k-\tau_{M}}^{k-\tau (k)-1}y^{T}(i)\; \sum_{i=k-\tau(k)}^{k-\tau_{m}-1}y^{T}(i) u^{T}(k) \Biggr]^{T},\end{aligned} \\& \Psi_{3} = \begin{pmatrix} S_{1}& U_{5}\\ *& U_{5} \end{pmatrix} ,\qquad \Psi_{4} = \begin{pmatrix} S_{2}& U_{6}\\ *& U_{6} \end{pmatrix} ,\qquad \Phi_{3} = \begin{pmatrix} S_{3}& U_{7}\\ *& U_{7} \end{pmatrix} ,\qquad \Phi_{4} = \begin{pmatrix} S_{4}& U_{8}\\ *& U_{8} \end{pmatrix} , \end{aligned}$$

and

$$ \varpi_{1} = \bigl[x^{T}(k) \eta^{T}(k) \bigr]^{T}, \qquad \Xi_{1} = \bigl[y^{T}(k) \zeta^{T}(k)\bigr]^{T}. $$

Next, in view of Schur complement [39], the RHS of (31) can be written as

$$\begin{gathered} \Psi+\Psi_{1}^{T}P_{1i}\Psi_{1}+\hat{ \sigma}^{2}\Psi _{2}^{T}Z_{1} \Psi_{2} +\frac{\check{\sigma}^{2}}{4}\Psi_{2}^{T}Z_{2}\Psi _{2}^{T}Z_{2}\Psi_{2} \\\quad\leq \begin{pmatrix} \Psi&\Psi_{1}^{T}P_{1i} &\hat{\sigma}\Psi_{2}^{T}Z_{1} &\frac {\check{\sigma}}{2}\Psi_{2}^{T}Z_{2}\\ * &-P_{1i} &0 &0\\ * &* &-Z_{1} &0\\ * &* &* &-Z_{2} \end{pmatrix} \end{gathered} $$

Similarly, for

$$\begin{gathered} \Phi+\Phi_{1}^{T}P_{2i}\Phi_{1}+\hat{ \tau}^{2}\Phi _{2}^{T}Z_{3} \Phi_{2} +\frac{\check{\tau}^{2}}{4}\Phi_{2}^{T}Z_{4} \Phi_{2}\\ \quad\leq \begin{pmatrix} \Phi&\Phi_{1}^{T}P_{2i} &\hat{\tau}\Phi_{2}^{T}Z_{3} &\frac {\check{\tau}}{2}\Phi_{2}^{T}Z_{4}\\ * &-P_{2i} &0 &0\\ * &* &-Z_{3} &0\\ * &* &* &-Z_{4} \end{pmatrix} \end{gathered} $$

Then, by using uncertainty description (2), (4) and procedure as in Lemma 2.6, we have

32 $$\begin{aligned}[b] &\Psi+\Psi_{1}^{T}P\Psi_{1}+\hat{ \sigma}^{2}\Psi_{2}^{T}Z_{1}\Psi _{2} +\frac{\check{\sigma}^{2}}{4}\Psi_{2}^{T}Z_{2}\Psi _{2}^{T}Z_{2}\Psi_{2}\\&\quad\leq \begin{pmatrix} \Psi&\tilde{\Pi}_{1}^{T}P_{1i} &\hat{\sigma}\tilde{\Pi }_{2}^{T}Z_{1} &\frac{\check{\sigma}}{2}\tilde{\Pi}_{2}^{T}Z_{2}\\ * &-P_{1i} &0 &0\\ * &* &-Z_{1} &0\\ * &* &* &-Z_{2} \end{pmatrix} +\operatorname{sym}\bigl(\tilde{M}N(k) \tilde{M}_{abc}\bigr) \\ &\quad\leq \begin{pmatrix} \Psi&\tilde{\Pi}_{1}^{T}P_{1i} &\hat{\sigma}\tilde{\Pi }_{2}Z_{1}^{T} &\frac{\check{\sigma}}{2}\tilde{\Pi}_{2}^{T}Z_{2}\\ * &-P_{1i} &0 &0\\ * &* &-Z_{1} &0\\ * &* &* &-Z_{2} \end{pmatrix} +\varepsilon^{-1} \tilde{M}\tilde{M}^{T}+\varepsilon\tilde {M}_{abc}^{T} \tilde{M}_{abc} , \end{aligned} $$

where

33 $$\begin{aligned}& \tilde{M}= \bigl[\begin{matrix}0_{1, 10n} &M^{T} & M^{T}& M^{T} \end{matrix}\bigr], \\& \tilde{M}_{abc}= [\begin{matrix}M_{A} & 0_{1,4n}& M_{B} & M_{C} & 0_{1, 7n}\end{matrix} ], \\& \Phi+\Phi_{1}^{T}P\Phi_{1}+\hat{ \tau}^{2}\Phi_{2}^{T}Z_{3}\Phi _{2}+\frac{\check{\tau}^{2}}{4}\Phi_{2}^{T}Z_{4} \Phi_{2}^{T}Z_{4}\Phi _{2} \\& \quad\leq \begin{pmatrix} \Phi&\breve{\Pi}_{1}^{T}P_{2i} &\hat{\tau}\breve{\Pi }_{2}^{T}Z_{3} &\frac{\check{\tau}}{2}\breve{\Pi}_{2}^{T}Z_{4}\\ * &-P_{2i} &0 &0\\ * &* &-Z_{3} &0\\ * &* &* &-Z_{4} \end{pmatrix} +\operatorname{sym}\bigl(\breve{M}N(k) \breve{M}_{def}\bigr) \\& \quad\leq \begin{pmatrix} \Phi&\breve{\Pi}_{1}^{T}P_{2i} &\hat{\tau}\breve{\Pi }_{2}Z_{3}^{T} &\frac{\check{\tau}}{2}\breve{\Pi}_{2}^{T}Z_{4}\\ * &-P_{2i} &0 &0\\ * &* &-Z_{3} &0\\ * &* &* &-Z_{4} \end{pmatrix} +\varepsilon_{1}^{-1} \breve{M}\breve{M}^{T}+\varepsilon_{1}\breve {M}_{def}^{T}\breve{M}_{def} , \end{aligned}$$

where

$$\begin{gathered} \breve{M}= \bigl[\begin{matrix}0_{1, 10n}& M^{T}& M^{T}& M^{T} \end{matrix}\bigr], \\ \breve{M}_{def}= [\begin{matrix}M_{D} & 0_{1,4n}& M_{E} & M_{F} & 0_{1, 7n} \end{matrix}].\end{gathered} $$

Hence if LMIs ((6), (7), (8), (9) hold, it is easy to get

34 $$\begin{aligned}& \Delta V(k)=\Delta V_{r1}(k)+\Delta V_{r2}(k) \\& \Delta V_{r1}(k)-(\mu-1)\Delta V_{r1}-v^{T}(k)Q v(k) \\& \quad{}+\Delta V_{r2}(k)-(\rho-1)\Delta V_{r2}-u^{T}(k)Q_{1}u(k) \leq 0 \\& \begin{aligned}[b]V(k+1)-V(k)\leq{}& \bigl[(\mu-1)+(\rho-1) \bigr]V(k)+u^{T}(k)Q_{1}u(k) \\ & +v^{T}(k)Q v(k) \\ \leq{}& \bigl[(\mu-1)+(\rho-1) \bigr]v(k)+\lambda _{Q_{1}}u^{T}(k)u(k) \\ & +\lambda_{Q}v^{T}(k)v(k),\end{aligned} \end{aligned}$$

where $\lambda_{Q_{1}}=\lambda_{\mathrm{max}}(Q_{1})$ and $\lambda _{Q}=\lambda_{\mathrm{max}}(Q)$. Simple computation gives

$$ V(k+1)\leq\mu V(k)+\rho V(k)+\lambda_{Q_{1}}u^{T}u(k)+ \lambda_{Q}v^{T}v(k). $$

Noticing $\mu\geq1$ and $\rho\geq1$, it follows that

35 $$\begin{aligned}[b] V(k)\leq{}&\mu^{k}V(0)+\rho^{k}V(0)+\lambda_{Q_{1}} \sum_{n=0}^{k-1}\mu^{k-n-1}u^{T}(n)u(n)+ \lambda_{Q}\sum_{n=0}^{k-1}\mu ^{k-n-1}v^{T}(n)v(n) \\ \leq{}&\mu^{k}V(0)+\rho^{k}V(0)+\mu^{k} \lambda_{Q_{1}}u+\mu ^{k}\lambda_{Q}v. \end{aligned} $$

Further, from (12), we can get

$$\begin{aligned} V(0) =&x^{T}(0)P_{1i}x(0)+y^{T}P_{2i}y(0)+ \sum_{i=-\sigma(0)}^{-1}\mu ^{-i-1} x^{T}(i)W x(i)+ \sum_{j=-\sigma_{M}+1}^{-\sigma_{M}} \sum_{i=j}^{-1}\mu^{-i-1} x^{T}(i)W x(i) \\ &{} +\sum_{i=k-\tau(0)}^{-1}\rho^{-i-1} y^{T}(i)W_{1} y(i)+ \sum_{j=-\tau_{M}+1}^{-\tau_{M}} \sum_{i=j}^{-1}\rho^{-i-1} y^{T}(i)W_{1} y(i) \\ &{} +\sum_{i=k-\sigma(0)}^{-1} \mu^{-i-1} g^{T}\bigl(x(i)\bigr) R g\bigl(x(i)\bigr)+ \sum_{j=-\sigma_{M}+1}^{-\sigma_{M}} \sum_{i=j}^{-1}\mu^{-i-1} g^{T}\bigl(x(i)\bigr)R g\bigl(x(i)\bigr) \\ &{} +\sum _{i=k-\tau(0)}^{-1}\rho^{-i-1} f^{T} \bigl(y(i)\bigr)R_{1} f\bigl(y(i)\bigr) + \sum_{j=-\tau_{M}+1}^{-\tau_{M}} \sum _{i=j}^{-1}\rho ^{-i-1} f^{T} \bigl(y(i)\bigr)R_{1} f\bigl(y(i)\bigr)\\ &{} +\sum _{j=-\sigma_{M}}^{-\sigma_{m}-1}\sum_{i=j}^{-1} \mu^{-i-1} x^{T}(i)S_{1}x(i) +\sum _{l=-\sigma_{M}}^{-\sigma_{m}-1}\sum_{j=l}^{-1}\sum_{i=j}^{-1} \mu^{-i-1} x^{T}(i)S_{2}x(i)\\ & {} +\sum _{j=-\tau_{(}M)}^{-\tau_{m}-1}\sum_{i=j}^{-1} \rho^{-i-1} y^{T}(i)S_{3}y(i)\\ &{}+\sum _{l=-\tau_{M}}^{-\tau_{m}-1}\sum_{j=l}^{-1} \sum_{i=j}^{-1}\rho^{-i-1} y^{T}(i)S_{4}y(i)\hat{\sigma}\sum _{j=-\sigma_{M}}^{-\sigma _{m}-1}\sum_{i=j}^{-1} \mu^{-i-1} \eta^{T}(i)Z_{1}\eta(i)\\ &{} + \frac {\check{\sigma}}{2}\sum_{l=-\sigma_{M}}^{-\sigma_{m}-1}\sum _{j=l}^{-1}\sum _{i=j}^{-1}\mu^{-i-1} \eta^{T}(i)Z_{2} \eta(i) +\hat{\tau}\sum_{j=-\tau_{(}M)}^{-\tau_{m}-1}\sum _{i=j}^{-1}\rho^{-i-1} \zeta^{T}(i)Z_{3}\zeta(i)\\ &{}+\frac{\check{\tau }}{2}\sum _{l=-\tau_{M}}^{-\tau_{m}-1} \sum_{i=j}^{-1} \rho^{-i-1} \zeta^{T}(i)Z_{4}\zeta(i). \end{aligned}$$

Letting

$$\begin{aligned}& P_{1}= L^{\frac{-1}{2}}P_{1i}L^{\frac{-1}{2}},\qquad P_{2}=L_{1}^{\frac{1}{2}}P_{2i}L_{1}^{\frac{-1}{2}};\qquad W_{3}=L^{\frac{-1}{2}}W_{1}L^{\frac{-1}{2}},\qquad W_{4}= L_{1}^{\frac{-1}{2}}W_{1}L_{1}^{\frac{-1}{2}}, \\ & R_{3}= L^{\frac{-1}{2}}R L^{\frac{-1}{2}}, \qquad R_{4}=L_{1}^{\frac {-1}{2}}R_{1}L^{\frac{-1}{2}},\qquad S_{11}= L^{\frac{-1}{2}}S_{1}L^{\frac{-1}{2}},\qquad S_{21}=L_{1}^{\frac{-1}{2}}S_{2}L^{\frac{-1}{2}}, \\ & S_{31}= L_{1}^{\frac{-1}{2}}S_{3}L_{1}^{\frac{-1}{2}},\qquad S_{41}=L_{1}^{\frac{-1}{2}}S_{4}L^{\frac{-1}{2}}, \end{aligned}$$

we obtain

$$\begin{aligned} V(0) &= x^{T}(0)L^{\frac{1}{2}}P_{1}L^{\frac {1}{2}}x(0)+y^{T}(0)L_{1}^{\frac{1}{2}}P_{2}L_{1}^{\frac {1}{2}}y(0)+ \sum_{i=-\sigma(0)}^{-1}\mu^{-i-1} x^{T}L^{\frac {1}{2}}(i)W_{3} L^{\frac{1}{2}}x(i) \\ & \quad {} + \sum _{j=-\sigma_{M}+1}^{-\sigma _{M}} \sum _{i=j}^{-1}\mu^{-i-1} x^{T}(i)L^{\frac{1}{2}}W_{3}L^{\frac {1}{2}} x(i) +\sum_{i=-\tau(0)}^{-1}\rho^{-i-1} y^{T}(i)L_{1}^{\frac {1}{2}}W_{4}L^{\frac{1}{2}}L_{1}^{\frac{1}{2}} y(i) \\ & \quad {} + \sum_{j=-\tau _{M}+1}^{-\tau_{M}} \sum _{i=j}^{-1}\rho^{-i-1} y^{T}(i)L_{1}^{\frac{1}{2}}W_{4} L_{1}^{\frac{1}{2}}y(i) +\sum_{i=k-\sigma(0)}^{-1} \mu^{-i-1} g^{T}\bigl(x(i)\bigr)L^{\frac{1}{2}} R_{3}L^{\frac{1}{2}} g\bigl(x(i)\bigr) \\ & \quad {} + \sum _{j=-\sigma_{M}+1}^{-\sigma_{M}} \sum_{i=j}^{-1} \mu^{-i-1} g^{T}\bigl(x(i)\bigr)L^{\frac{1}{2}}R_{3}L^{\frac{1}{2}} g\bigl(x(i)\bigr) +\sum_{i=k-\tau(0)}^{-1} \rho^{-i-1} f^{T}\bigl(y(i)\bigr)L_{1}^{\frac {1}{2}}R_{4}L_{1}^{\frac{1}{2}} f\bigl(y(i)\bigr) \\ & \quad {} + \sum_{j=-\tau_{M}+1}^{-\tau_{M}} \sum _{i=j}^{-1}\rho^{-i-1} f^{T}\bigl(y(i)\bigr)L_{1}^{\frac {1}{2}}R_{4}L_{1}^{\frac{1}{2}} f\bigl(y(i)\bigr) +\sum_{j=-\sigma_{M}}^{-\sigma_{m}-1}\sum _{i=j}^{-1}\mu^{-i-1} x^{T}(i)L^{\frac{1}{2}}S_{11}L^{\frac{1}{2}}x(i) \\ & \quad {} +\sum _{l=-\sigma _{M}}^{-\sigma_{m}-1}\sum _{j=l}^{-1} \sum_{i=j}^{-1}\times\mu^{-i-1} x^{T}(i)L^{\frac{1}{2}}S_{21}L^{\frac{1}{2}}x(i)+\sum_{j=-\tau_{(}M)}^{-\tau_{m}-1}\sum _{i=j}^{-1}\rho^{-i-1} y^{T}(i)L_{1}^{\frac{1}{2}}S_{31}L_{1}^{\frac{1}{2}}y(i) \\ & \quad {} + \sum_{l=-\tau_{M}}^{-\tau_{m}-1}\sum _{j=l}^{-1}\sum_{i=j}^{-1} \rho ^{-i-1} y^{T}(i)L_{1}^{\frac{1}{2}}S_{41}L_{1}^{\frac {1}{2}}y(i)+ \hat{\sigma}\sum_{j=-\sigma_{M}}^{-\sigma_{m}-1}\sum _{i=j}^{-1}\mu^{-i-1} \eta^{T}(i)Z_{1} \eta(i) \\ & \quad {} +\frac{\check{\sigma }}{2}\sum_{l=-\sigma_{M}}^{-\sigma_{m}-1} \sum_{j=l}^{-1}\sum _{i=j}^{-1}\mu^{-i-1} \eta^{T}(i)Z_{2} \eta(i)+\hat{\tau}\sum_{j=-\tau_{(}M)}^{-\tau_{m}-1}\sum _{i=j}^{-1}\rho^{-i-1} \zeta^{T}(i)Z_{3}\zeta(i) \\ & \quad {} +\frac{\check{\tau}}{2} \sum _{l=-\tau_{M}}^{-\tau_{m}-1}\sum_{i=j}^{-1} \rho^{-i-1} \zeta ^{T}(i)Z_{4}\zeta(i) \\ & \leq \lambda_{P_{1}}x^{T}(0)L x(0)+\lambda_{P_{2}}y^{T}(0)L y(0)+\lambda _{W_{3}}\sum_{i=-\sigma(0)}^{-1} \mu^{-i-1} x^{T}L x(i) \\ & \quad {} + \lambda _{W_{3}}\sum _{j=-\sigma_{M}+1}^{-\sigma_{M}} \sum_{i=j}^{-1} \mu^{-i-1} x^{T}(i) L x(i) +\lambda_{W_{4}}\sum_{i=-\tau(0)}^{-1} \rho ^{-i-1} y^{T}(i)L_{1}y(i) \\ & \quad {} +\lambda_{W_{4}} \sum_{j=-\tau_{M}+1}^{-\tau _{M}} \sum _{i=j}^{-1}\rho^{-i-1} y^{T}(i)L_{1} y(i) +\hat{g}^{2}\lambda_{R_{3}}\sum _{i=k-\sigma(0)}^{-1}s\mu^{-i-1} x^{T}(i)L x(i) \\ & \quad {} +\hat{g}^{2} \lambda_{R_{3}}\sum_{j=-\sigma_{M}+1}^{-\sigma _{M}} \sum _{i=j}^{-1}\mu^{-i-1} x^{T}(i)L x(i)+\hat{f}^{2}\lambda_{R_{4}}\sum _{i=k-\tau (0)}^{-1}\rho^{-i-1} y^{T}(i)L_{1}y(i) \\ & \quad {} + \hat{f}^{2}\lambda{R_{4}}\sum _{j=-\tau_{M}+1}^{-\tau _{M}} \sum_{i=j}^{-1} \rho^{-i-1} y^{T}(i)L_{1} y(i)+\lambda_{S_{11}} \hat{\sigma}\sum_{j=-\sigma_{M}}^{-\sigma_{m}-1}\sum _{i=j}^{-1}\mu^{-i-1} x^{T}(i)x(i) \\ & \quad {} + \lambda_{S_{21}}\hat{\sigma}\sum_{l=-\sigma _{M}}^{-\sigma_{m}-1} \sum_{j=l}^{-1} \sum_{i=j}^{-1} \mu^{-i-1} x^{T}(i)x(i)+\lambda _{S_{31}}\hat{\tau}\sum _{j=-\tau_{(}M)}^{-\tau_{m}-1}\sum _{i=j}^{-1}\rho^{-i-1} y^{T}(i)y(i) \\ & \quad {} + \lambda_{S_{41}}\hat{\tau}\sum_{l=-\tau_{M}}^{-\tau_{m}-1} \sum_{j=l}^{-1}\sum _{i=j}^{-1}\rho ^{-i-1}y^{T}(i)y(i) +\lambda_{Z_{1}}\hat{\sigma}\sum_{j=-\sigma_{M}}^{-\sigma _{m}-1} \sum_{i=j}^{-1}\mu^{-i-1} \eta^{T}(i)\eta(i) \\ & \quad {} +\lambda _{Z_{2}}\frac{\check{\sigma}}{2}\sum _{l=-\sigma_{M}}^{-\sigma _{m}-1}\sum _{j=l}^{-1}\sum_{i=j}^{-1} \mu^{-i-1} \eta^{T}(i)\eta(i) +\lambda_{Z_{3}}\hat{\tau}\sum_{j=-\tau_{(}M)}^{-\tau_{m}-1} \sum_{i=j}^{-1}\rho^{-i-1} \zeta^{T}(i)\zeta(i) \\ & \quad {} +\lambda_{Z_{4}}\frac {\check{\tau}}{2} \sum _{l=-\tau_{M}}^{-\tau_{m}-1}\sum _{i=j}^{-1}\rho^{-i-1} \zeta ^{T}(i)\zeta(i) \\ & \leq \biggl[\lambda_{P_{1}}+\biggl(\mu^{\sigma_{M}-1} \sigma_{M}+\mu ^{\sigma_{M}}\frac{\hat{\sigma}+\sigma_{m}-1}{2}\biggr) \bigl(\lambda _{W_{3}}+\hat{f}^{2}\lambda_{R_{3}}\bigr) + \lambda_{S_{11}}\mu^{\sigma_{M}-1}\frac{\check{\sigma }}{2} \\ & \quad {} +\lambda_{S_{21}} \mu^{\sigma_{M}-1} \biggl[\biggl(\frac{1}{12}\sigma _{M}(\sigma_{M}+1) (2\sigma_{M}+1)- \sigma_{m} (\sigma_{m}+1) (2\sigma_{m}+1)\biggr)- \frac{\check{\sigma}}{4} \biggr] \biggr]\eta_{1} \\ & \quad {} + \biggl[ \lambda_{P_{2}}+\biggl(\rho^{\tau_{M}-1}\tau_{M}+\rho ^{\tau_{M}}\frac{\hat{\tau}+\tau_{m}-1}{2}\biggr)\bigl(\tau_{W_{4}}+\hat{g}^{2} \lambda_{R_{4}}\bigr) +\lambda_{S_{31}}\rho^{\tau_{M}-1} \frac{\check{\tau}}{2} \\ & \quad {} +\lambda _{S_{41}}\rho^{\tau_{M}-1} \biggl[\biggl( \frac{1}{12}\tau_{M}(\tau _{M}+1) (2 \tau_{M}+1)-\tau_{m} (\tau_{m}+1) (2 \tau_{m}+1)\biggr) -\frac{\check{\tau}}{4} \biggr] \biggr]\eta_{2} \\ & \quad {} + \biggl[\lambda _{Z_{1}}\mu^{\sigma_{M}-1}\frac{\hat{\sigma}\check{\sigma }}{2} \\ & \quad {} +\lambda_{Z_{2}} \mu^{\sigma_{M}-1} \check{\sigma} \biggl[\biggl(\frac{1}{12} \sigma_{M}(\sigma_{M}+1) (2\sigma _{M}+1)- \sigma_{m} (\sigma_{m}+1) (2\sigma_{m}+1)\biggr)-\frac{\check{\sigma}}{8} \biggr] \biggr]\kappa_{1} \\ & \quad {} + \biggl[ \lambda_{Z_{3}}\rho^{\tau_{M}-1}\frac{\hat{\tau}\check{\tau }}{2} \\ & \quad {} + \lambda_{Z_{4}}\rho^{\tau_{M}-1} \check{\tau} \biggl[\biggl( \frac{1}{12}\tau_{M}(\tau_{M}+1) (2\tau _{M}+1)-\tau_{m} (\tau_{m}+1) (2 \tau_{m}+1)\biggr)-\frac{\check{\tau}}{8} \biggr] \biggr] \kappa_{2}, \end{aligned}$$

where $\lambda_{P_{1}}= \lambda_{\max}(P_{1})$, $\lambda_{P_{2}}= \lambda_{\max}(P_{2})$, $\lambda^{P_{1}}= \lambda_{\min}(P_{1})$, $\lambda^{P_{2}}= \lambda_{\min}(P_{2})$, $\lambda_{W_{3}}= \lambda _{\max}(W_{3})$, $\lambda_{W_{4}}= \lambda_{\max}(W_{4})$, $\lambda _{R_{3}}= \lambda_{\max}(R_{3})$, $\lambda_{R_{4}}= \lambda_{\max }(R_{4})$, $\lambda_{S_{1}}= \lambda_{\max}(S_{11})$, $\lambda _{S_{2}}= \lambda_{\max}(S_{21})$, $\lambda_{S_{3}}= \lambda_{\max }(S_{31})$, $\lambda_{S_{4}}= \lambda_{\max}(S_{41})$, $\lambda _{Z_{1}}= \lambda_{\max}(Z_{1})$, $\lambda_{Z_{2}}= \lambda_{\max }(Z_{2})$, $\lambda_{Z_{3}}= \lambda_{\max}(Z_{3})$, $\lambda _{Z_{4}}= \lambda_{\max}(Z_{4})$.

On the other hand, from (12), we can obtain that

36 $$\begin{aligned}[b] V(k)&\geq x^{T}(k)P_{1i}x(k)+y^{T}(k)P_{2i}y(k) \\ &\geq x^{T}(k)L^{\frac{1}{2}}P_{1}L^{\frac {1}{2}}x(k)+y^{T}(k)L_{1}^{\frac{1}{2}}P_{2}L_{1}^{\frac {1}{2}}y(k) \\ &\geq \lambda^{P_{1}}x^{T}(k)L x(k)+y^{T}(k)L_{1}^{\frac {1}{2}}P_{2}L_{1}^{\frac{1}{2}}y(k) \\ &\geq M^{*}\bigl[x^{T}(k)L x(k)+y^{T}(k)L_{1}y(k) \bigr]. \end{aligned} $$

Put $M^{*}=\min\{\lambda^{P_{1}},\lambda^{P_{2}}\}$.

From (35) to (36), we get

$$\begin{aligned} x^{T}(k)L x(k)+y^{T}(k)L_{1}y(k) &< \frac{(\psi_{1}\eta_{1}+\psi _{2}\kappa+\lambda_{Q_{1}}u)\mu^{k}+(\psi_{2}\eta_{2}+\psi _{3}\kappa_{1}+\lambda_{Q}v)\rho^{k} }{M^{*}} \\ &< \frac{(\psi_{1}\eta+\psi_{2}\kappa+\lambda_{Q_{1}}u)\mu ^{k}+(\psi_{2}\eta+\psi_{3}\kappa_{1}+\lambda_{Q}v)\rho^{k}}{M^{*}}.\end{aligned} $$

Therefore, from (11), we get that

$$ x^{T}(k)L x(k)+y^{T}(k)L_{1}y(k)< \chi, \quad\forall k \in \{1, 2, ,\ldots, N\}. $$

Then, using Definition 2.2, DNN (1), (3) is robustly finite-time bounded. □

### Remark 3.2

Leakage time delay in the stabilizing negative feedback term has a tendency to destabilize a system. The term $x(k-\gamma_{1})$, $y(k-\gamma_{2})$ in system (1) and (3) corresponds to a stabilizing negative feedback of the system which acts instantaneously with time delay. The term is variously known as leakage (or forgetting) term which is considered as a time delay.

## Robust finite-time passivity

In this subsection, we focus on the robust finite-time passivity of DNN (1), (3) with output (2), (4). In order to deal this, we introduce $I=2g^{T}(k)u(k)+u^{T}(k)Qu(k)$ and $J=2h^{T}(k)v(k)+v^{T}(k)Q_{1}v(k)$.

### Theorem 4.1

*Under Assumptions* I *and* II, *for given scalars* $\mu>1$, $\rho>1$, $\tau_{m}$, $\tau_{M}$, $\sigma_{m}$, $\sigma_{M}$, *DNN model* (1), (3) *is robustly finite*-*time passive with respect to* $(\eta_{1}, \eta_{2}, \eta, \chi, L, Q, L_{1}, Q_{1}, u, v)$, *if there exist symmetric positive definite matrices* $P_{1i}$, $P_{2i}$, *W*, $W_{1}$, $R_{1}$, *R*, $S_{1}$, $S_{2}$, $S_{3}$, $S_{4}$, $Z_{1}$, $Z_{2}$, $Z_{3}$, $Z_{4}$, *matrices* $U_{1}$, $U_{2}$, $U_{3}$, $U_{4}$, $U_{5}$, $U_{6}$, $U_{7}$, $U_{8}$, *positive diagonal matrices* $H_{1}$, $H_{2}$, $H_{3}$, $H_{4}$ *and positive scalars* $\lambda^{P_{1}}$, $\lambda^{P_{2}}$, $\lambda _{P_{1}}$, $\lambda_{P_{2}}$, $\lambda_{W_{3}}$, $\lambda_{W_{4}}$, $\lambda_{R_{3}}$, $\lambda_{R_{4}}$, $\lambda_{S_{1}}$, $\lambda _{S_{2}}$, $\lambda_{S_{3}}$, $\lambda_{S_{4}}$, $\lambda_{Z_{1}}$, $\lambda_{Z_{2}}$, $\lambda_{Z_{3}}$, $\lambda_{Z_{4}}$, *ϵ*, $\epsilon^{*}$, $P_{1i}=P_{1}(r_{k})$, $P_{2i}=P_{2}(r_{k})$, $\bar {P}_{1i}= \sum_{j=1}^{N}\Pi_{ij}P_{1j}$, $\bar{P}_{2i}= \sum_{j=1}^{N}\Pi_{ij}P_{2j}$, *ω*, $\omega_{1}$ *such that the following LMIs* (8), (9) *hold for* $r=1, 2, 3, 4$:

37 $$\begin{aligned}& \begin{pmatrix} \tilde{\Lambda}_{r}& \tilde{\Pi}_{1}^{T}P_{1i}& \hat{\sigma }\tilde{\Pi}_{2}^{T}Z_{1}& \frac{\check{\sigma}}{2}\tilde{\Pi }_{2}^{T}Z_{2}& 0& \epsilon\tilde{M}^{T}_{ABC}\\ *& -P_{1i}& 0& 0& M& 0\\ *& *& -Z_{1}& 0& M& 0\\ *& *& *& -Z_{2}& M& 0\\ *& *& *& *& -\epsilon I& 0\\ *& *& *& *& *& -\epsilon I \end{pmatrix} < 0 ,\quad r=1,2, \end{aligned}$$

38 $$\begin{aligned}& \begin{pmatrix} \breve{\Lambda}_{r}& \breve{\Pi}_{1}^{T}P_{2i}& \hat{\tau}\breve {\Pi}_{2}^{T}Z_{3}& \frac{\check{\tau}}{2}\breve{\Pi }_{2}^{T}Z_{4}& 0& \epsilon^{*}\breve{M}^{T}_{DEF}\\ *& -P_{2i}& 0& 0& M& 0\\ *& *& -Z_{1}& 0& M& 0\\ *& *& *& -Z_{2}& M& 0\\ *& *& *& *& -\epsilon^{*} I& 0\\ *& *& *& *& *& -\epsilon^{*} I \end{pmatrix} < 0 ,\quad r=3,4, \end{aligned}$$

39 $$\begin{aligned}& \psi_{1}\eta_{1}+\psi_{2}\kappa+ \mu^{-T}u+\psi_{3}\eta_{2}+\psi _{4} \kappa_{1}+\rho^{-T_{1}}v < M^{*}\chi\mu^{-T} \rho^{-T_{1}}, \end{aligned}$$

*where*

$$\begin{gathered} \tilde{\Lambda}_{r}= \begin{pmatrix} \tilde{\Pi}_{1,1,i}& 0& 0& 0& 0& 0& 0& 0& 0& 0\\ *& \tilde{\Pi}_{2,2,i}& 0& 0& 0& G_{2}H_{1}& 0& \tilde{\Pi}_{r2}& \tilde{\Pi}_{r3}& 0\\ *& *& \tilde{\Pi}_{3,3,i}& \tilde{\Pi}_{3,4,i}& \mu^{\sigma _{m}+1}U_{1}^{T}& 0& 0& 0& 0& 0\\ *& *& *& \tilde{\Pi}_{4,4,i}& \tilde{\Pi}_{4,5,i}& 0& G_{2}H_{2}& 0& 0& 0\\ *& *& *& *& \tilde{\Pi}_{5,5,i}& 0& 0& 0& 0& 0\\ *& *& *& *& *& \tilde{\Pi}_{6,6,i}& 0& 0& 0& -B_{g^{*}}^{T}\\ *& *& *& *& *& *& \tilde{\Pi}_{7,7,i}& 0& 0& -C_{g^{*}}^{T}\\ *& *& *& *& *& *& *& \tilde{\Pi}_{8,8,i}& \tilde{\Pi}_{8,9,i}& 0\\ *& *& *& *& *& *& *& *& \tilde{\Pi}_{9,9,i}& 0\\ *& *& *& *& *& *& *& *& *& -\omega I \end{pmatrix} ,\\\quad\quad r=1,2, \\ \breve{\Lambda}_{r}= \begin{pmatrix} \breve{\Pi}_{1,1,i}& 0& 0& 0& 0& 0& 0& 0& 0& 0\\ *& \breve{\Pi}_{2,2,i}& 0& 0& 0& F_{4}H_{3}& 0& \breve{\Pi}_{r2}& \breve{\Pi}_{r3}& 0\\ *& *& \breve{\Pi}_{3,3,i}& \breve{\Pi}_{3,4,i}& \rho^{\tau _{m}+1}U_{3}^{T}& 0& 0& 0& 0& 0\\ *& *& *& \breve{\Pi}_{4,4,i}& \breve{\Pi}_{4,5,i}& 0& G_{4}H_{4}& 0& 0& 0\\ *& *& *& *& \breve{\Pi}_{5,5,i}& 0& 0& 0& 0& 0\\ *& *& *& *& *& \breve{\Pi}_{6,6,i}& 0& 0& 0& -E_{h}^{T}\\ *& *& *& *& *& *& \breve{\Pi}_{7,7,i}& 0& 0& -F_{h}^{T}\\ *& *& *& *& *& *& *& \breve{\Pi}_{8,8,i}& \breve{\Pi}_{8,9,i}& 0\\ *& *& *& *& *& *& *& *& \breve{\Pi}_{9,9,i}& 0\\ *& *& *& *& *& *& *& *& *& -\omega_{1}I \end{pmatrix} ,\\\quad\quad r=3,4\end{gathered} $$

*and the parameters are defined in the above theorem*.

### Proof

The proof is followed from the theorem above by choosing $u = \omega \mu^{-T}$ in *I* and $v = \omega_{1}\rho^{-T_{1}}$ in *J*. Using similar lines of (34), it follows that

$$\begin{gathered} \Delta V_{r1}(k)-(\mu-1)V_{r1}(k)-2g^{T}(k)u(k)- \omega\mu ^{-T}u^{T}(k)u(k) \\ \quad{}+\Delta V_{r2}(k)-(\rho-1)V_{r2}(k)-2h^{T}(k)v(k)- \omega_{1}\rho ^{-T_{1}}v^{T}(k)v(k)\leq0, \\\begin{aligned} V(k+1)-V(k)\leq{}& (\mu-1)V_{r1}(k)+(\rho -1)V_{r2}(k)+2g^{T}(k)u(k)+2h^{T}(k)v(k) \\ & + \omega\mu^{-T}u^{T}(k)u(k)+\omega_{1}\mu^{-T_{1}}v^{T}(k)v(k).\end{aligned}\end{gathered} $$

By simple computation,

$$\begin{aligned} v(k)\leq{}&\mu^{k}v_{r1}(0)+\rho^{k}v_{r2}(0)+2 \sum_{i=0}^{k-1}\mu ^{k-i-1}g^{T}(i)u(i)+2 \sum_{i=0}^{k-1}\rho^{k-i-1}h^{T}(i)v(i) \\ & +w\mu^{-T}\sum_{i=0}^{k-1} \mu^{k-i-1}u^{T}(i)u(i)+w_{1}\mu ^{-T_{1}}\sum _{i=0}^{k-1}\rho^{k-i-1}v^{T}(i)v(i).\end{aligned} $$

Under the zero initial condition and noticing $v(k)\geq0$ for all $k \in \{1, 2 , 3, \ldots,N\}$, we have

$$\begin{aligned} 2\sum_{i=0}^{k-1}\mu^{k-i-1}g^{T}(i)u(i)+2 \sum_{i=0}^{k-1}\rho ^{k-i-1}h^{T}(i)v(i) \geq{}&{-} \omega\mu^{-T}\sum_{i=0}^{k-1} \mu ^{k-i-1}u^{T}(i)u(i) \\ & -\omega_{1}\mu^{-T_{1}}\sum_{i=0}^{k-1} \rho^{k-i-1}v^{T}(i)v(i). \end{aligned}$$

Noticing that $\mu, \rho\geq1$, we have

40 $$\begin{aligned}[b] 2 \Biggl[\sum_{k=0}^{T} \mu^{T-k}g^{T}(k)u(k)+\sum_{k=0}^{T_{1}} \rho ^{T_{1}-k}h^{T}(k)v(k) \Biggr]\geq{}& {-}\omega\mu^{-T} \sum_{k=0}^{T}\mu^{T-k}u^{T}(k)u(k) \\ & -\omega_{1}\rho^{-T_{1}}\sum_{k=0}^{T_{1}} \rho^{T_{1}-k}v^{T}(k)v(k). \end{aligned} $$

Let $\gamma^{*}=\max\{\omega, \omega_{1}\}$

$$\begin{gathered} 2 \Biggl[\sum_{k=0}^{T} \mu^{T-k}g^{{*}^{T}}(k)u(k)+\sum_{k=0}^{T_{1}} \rho^{T_{1}-k}h^{T}(k)v(k) \Biggr]\\\quad\geq \gamma ^{*} \Biggl[-\mu^{-T}\sum_{k=0}^{T} \mu^{T-k}g^{{*}^{T}}(k)u(k)-\rho ^{-T_{1}} \Biggr].\end{gathered} $$

Therefore, from (40), it is easy to get the inequality in Definition 2.2. Hence it can be concluded that DNN model (1), (3) is robustly finite-time passive. This completes the proof. □

### Remark 4.2

If leakage terms $\gamma_{1}$ and $\gamma_{2}$ become zero, then the neural networks system (1)-(4) is

$$\begin{gathered} x(k+1) = A\bigl(r(k)\bigr)x(k)+B\bigl(r(k)\bigr)f\bigl(y(k)\bigr)+C\bigl(r(k) \bigr)f\bigl(y\bigl(k-\tau(k)\bigr)\bigr)+u(k), \\ g^{*}(k) = B_{g^{*}}\bigl(r(k)\bigr)f\bigl(y(k) \bigr)+C_{g^{*}}f\bigl(y\bigl(k-\tau(k)\bigr)\bigr), \\ x(k)= \phi(k)\quad \text{for every } k \in [-\tau_{M},0], \\ y(k+1) = D\bigl(r(k)\bigr)y(k)+E\bigl(r(k)\bigr)g\bigl(x(k)\bigr)+F\bigl(r(k) \bigr)g\bigl(x\bigl(k-\sigma(k)\bigr)\bigr)+v(k), \\ h(k) = E_{h}\bigl(r(k)\bigr)g\bigl(x(k)\bigr)+F_{h}g\bigl(x \bigl(k-\sigma(k)\bigr)\bigr), \\ y(k)= \psi(k) \quad\text{for every } k \in [-\sigma_{M},0].\end{gathered} $$

## Numerical simulation

In this section, we present one numerical example with its simulations to guarantee the superiority and validity of our theoretical results.

### Example 5.1

Consider two-dimensional robust finite-time passivity Markovian jumping discrete-time BAM neural networks with (1)-(4) with $x(k) = (x_{1}(k),x_{2}(k))^{T}$, $y(k) = (y_{1}(k),y_{2}(k))^{T}$; $r(k)$ denotes right-continuous Markovian chains taking values in $\mathfrak{M}=\{1,2\}$ with generators

$$\begin{gathered} A= \begin{pmatrix} 0.05& 0\\ 0& 0.04 \end{pmatrix} ,\qquad B = \begin{pmatrix} -0.02& 0.08\\ 0.09& 0.03 \end{pmatrix} ,\qquad C= \begin{pmatrix} 0.06& 0.03\\ -0.08& 0.05 \end{pmatrix} , \\ D= \begin{pmatrix} 0.03& 0\\ 0& 0.02 \end{pmatrix} ,\qquad E= \begin{pmatrix} 0.01& -0.1\\ 0.02& 1.1 \end{pmatrix} ,\qquad F= \begin{pmatrix} 1& 0.03\\ 0.03& -0.04 \end{pmatrix} , \\ G_{1}= \begin{pmatrix} -8& 2\\ 2& 1 \end{pmatrix} ,\qquad G_{2}= \begin{pmatrix} 1& 5\\ 4.23& -4.3 \end{pmatrix} ,\\ F_{1}= \begin{pmatrix} 6& 5\\ 5& 0.6 \end{pmatrix} ,\qquad F_{2}= \begin{pmatrix} 5& 0.9\\ 2& 8 \end{pmatrix} ,\\ G_{3}= \begin{pmatrix} -4& 0.9\\ 2& 7 \end{pmatrix} ,\qquad G_{4}= \begin{pmatrix} 2& 4\\ 6& -2 \end{pmatrix} ,\\ F_{3}= \begin{pmatrix} 8& 0.9\\ 6.67& 0.8 \end{pmatrix} ,\qquad F_{4}= \begin{pmatrix} 1.9& 0.7\\ 0.9& 2.1 \end{pmatrix} ,\\ M_{A}= \begin{pmatrix} 0.3& 0\\ 0& 0.3 \end{pmatrix} ,\qquad M_{B}= \begin{pmatrix} 0.05& 0\\ 0& 0.05 \end{pmatrix} ,\qquad M_{C}= \begin{pmatrix} 0.07& 0\\ 0& 0.07 \end{pmatrix} ,\\ M_{D}= \begin{pmatrix} 0.2& 0\\ 0& 0.2 \end{pmatrix} ,\qquad M_{E}= \begin{pmatrix} 0.3& 0\\ 0& 0.3 \end{pmatrix} ,\qquad M_{F}= \begin{pmatrix} 0.8& 0\\ 0& 0.8 \end{pmatrix} .\end{gathered} $$

Leakage delay is defined as $\gamma_{1} = \gamma_{2}= 5$, and the scalars are as follows: $\mu=5$; $\rho=9$; $\eta_{1}=9$; $\eta _{2}=30$; $\eta=8$; $\xi=9$; $L=5$; $Q=4$; $L_{1}=3$; $Q_{1}=8$; $u=10$; $v=30$. Lower bounds and upper bounds of finite-time passivity BAM neural networks system (1)-(4) are $\tau _{M}=50$, $\tau_{m}=8$, $\sigma_{m}=5$, and $\sigma_{M}=30$.

Now, take the activation functions as follows:

$$ f_{1}\bigl(y(k)\bigr)=g_{1}\bigl(y(k)\bigr)= \begin{pmatrix} \tanh(-0.8y)\\ \tanh(-0.8y) \end{pmatrix} ,\qquad f_{2}\bigl(x(k) \bigr)=g_{2}\bigl(x(k)\bigr)= \begin{pmatrix} \tanh(-0.8x)\\ \tanh(-0.8x) \end{pmatrix} . $$

Now, the feasible solutions are as follows:

$$\begin{aligned}& P_{11} = 10^{3} \times \begin{pmatrix} 2.3666& -0.1023\\ -0.1023& 2.0944 \end{pmatrix} ,\qquad P_{21} = \begin{pmatrix} 5.8034& -0.1288\\ -0.1288& 6.1511 \end{pmatrix} ,\\& P_{12} = \begin{pmatrix} 3.3526& 0\\ 0& 3.3526 \end{pmatrix} ,\qquad P_{22} = \begin{pmatrix} 6.2695 & 0\\ 0& 6.4310 \end{pmatrix} ,\\& \bar{P}_{11} = \begin{pmatrix} 3.3526 & 0\\ 0& 3.3526 \end{pmatrix} ,\qquad \bar{P}_{21} = \begin{pmatrix} 3.3526 & 0\\ 0& 3.3526 \end{pmatrix} , \\& \bar{P}_{12} = \begin{pmatrix} 3.3526 & 0\\ 0& 3.3526 \end{pmatrix} ,\qquad \bar{P}_{22} = \begin{pmatrix} 3.3526 & 0\\ 0& 3.3526 \end{pmatrix} ,\\& W = \begin{pmatrix} 4.8570& -0.3754\\ -0.3754& 3.7776 \end{pmatrix} ,\qquad W_{1}= 10^{5}\times \begin{pmatrix} 0.4250& 0.0005\\ 0.0005& 0.3395 \end{pmatrix} ,\\& R= \begin{pmatrix} 1.7080& -0.0980\\ -0.0980& 1.7150 \end{pmatrix} ,\qquad R_{1}= 10^{6} \begin{pmatrix} 0.1283& -0.0021\\ -0.0021& 0.1222 \end{pmatrix} , \\& S_{1}= \begin{pmatrix} 240.2276& 29.0528\\ 29.0528& 317.4281 \end{pmatrix} ,\qquad S_{2}= \begin{pmatrix} 448.2930& 49.5842\\ 49.5842& 579.8507 \end{pmatrix} ,\\& S_{3}= \begin{pmatrix} 2.1983& 0.0489\\ 0.0489& 2.0885 \end{pmatrix} ,\qquad S_{4}= \begin{pmatrix} 2.6581& 0.0369\\ 0.0369& 2.5750 \end{pmatrix} ,\\& Z_{1} \begin{pmatrix} 0.7935& -0.0290\\ -0.0290& 0.8393 \end{pmatrix} ,\qquad Z_{2}=10^{3} \times \begin{pmatrix} 0.0487& -0.0023\\ -0.0023& 0.0434 \end{pmatrix} , \\& Z_{3}= \begin{pmatrix} 0.8915& 0.0182\\ 0.0182& 0.9041 \end{pmatrix} ,\qquad Z_{4}= \begin{pmatrix} 0.4589& -0.0047\\ -0.0047& 0.4605 \end{pmatrix} ,\\& U_{1}= \begin{pmatrix} 0.5777& -0.0141\\ -0.0141& 0.6023 \end{pmatrix} ,\qquad U_{2}= \begin{pmatrix} 0.0100& -0.0013\\ -0.0013& 0.0110 \end{pmatrix} ,\\& U_{3}= \begin{pmatrix} 0.8905& -0.1991\\ -0.1991& 0.8906 \end{pmatrix} ,\qquad U_{4}= \begin{pmatrix} 0.1132& -0.0011\\ -0.0011& 0.1111 \end{pmatrix} , \\& U_{5}= \begin{pmatrix} 2.2529& 0.0058\\ 0.0058& 2.4217 \end{pmatrix} ,\qquad U_{6}= \begin{pmatrix} 0.1111& 0.0105\\ 0.0105& 0.1469 \end{pmatrix} ,\\& U_{7}= \begin{pmatrix} 0.1740& 0.0144\\ 0.0144& 0.1768 \end{pmatrix} ,\quad\quad U_{8}= \begin{pmatrix} 0.1214& 0.0037\\ 0.0037& 0.1112 \end{pmatrix} ,\\& \epsilon=\epsilon1 =10^{3} \times (3.3526) ,\qquad H_{1}= \begin{pmatrix} 383.2021& 0\\ 0& 456.5170 \end{pmatrix} , \\& H_{2}= \begin{pmatrix} 559.7824& 0\\ 0& 319.0622 \end{pmatrix} ,\qquad H_{3}= \begin{pmatrix} 922.4418& 0\\ 0& 841.9595 \end{pmatrix} ,\\& H_{4}= \begin{pmatrix} 352.9877 & 0\\ 0& 400.7669 \end{pmatrix} . \end{aligned}$$

The trajectory of finite-time passivity BAM neural networks system (1)-(4) is shown in Figure 2.

**Figure 1:**
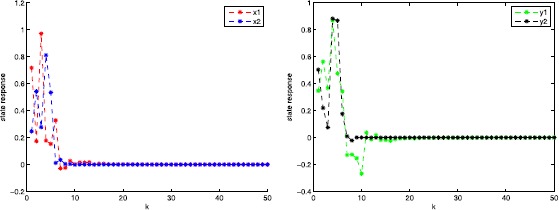
**The state response** $\pmb{x(k)}$ **,** $\pmb{y(k)}$ **of (** **1** **)-(** **4** **) with leakage delay.**

**Figure 2:**
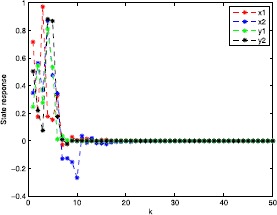
**State trajectories of finite-time passivity BAM neural networks (** **1** **)-(** **4** **).**

**Figure 3:**
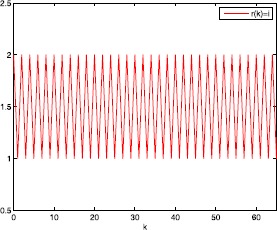
$\pmb{r(k)}$ **denotes Markovian jump of (** **1** **)-(** **4** **).**

According to Theorem 4.1, we can obtain that system (1)-(4) with the above given parameters is exponentially stable. With the help of Lyapunov functions and state trajectories $x_{1}(k)$, $x_{2}(k)$, $y_{1} (k)$, $y_{2}(k)$, the above finite-time passivity BAM neural networks are depicted in Figures 1, 2 and 3.

The performance of Markovian jumping for system (1)-(4) is given in Figure 3.

## Conclusion

Passivity result for uncertain discrete-time Markovian jumping BAM neural networks with leakage delay has been investigated. By using the Lyapunov theory together with zero inequalities, convex combination and reciprocally convex combination approaches, the finite-time boundedness and passivity are derived in terms of LMI which can be easily verified via the LMI toolbox. Leakage delay has been considered as a time-varying delay. Utilizing the reciprocal convex technique, conservatism of the proposed criteria has been reduced significantly. A numerical example has been provided to illustrate the effectiveness of the results and their improvement over the existing results.

**Table 1:** **Optimal values of** ***χ*** **for different** $\pmb{\tau_{M}}$ **and** $\pmb{\sigma_{M}}$

$\tau_{M}$:	12	14	16	18	20	22
$\sigma_{M}$:	10	12	14	16	18	20
*χ*	5.3	8	10.6	12	13.89	15.04
